# Lipidome changes due to improved dietary fat quality inform cardiometabolic risk reduction and precision nutrition

**DOI:** 10.1038/s41591-024-03124-1

**Published:** 2024-07-11

**Authors:** Fabian Eichelmann, Marcela Prada, Laury Sellem, Kim G. Jackson, Jordi Salas Salvadó, Cristina Razquin Burillo, Ramon Estruch, Michael Friedén, Frederik Rosqvist, Ulf Risérus, Kathryn M. Rexrode, Marta Guasch-Ferré, Qi Sun, Walter C. Willett, Miguel Angel Martinez-Gonzalez, Julie A. Lovegrove, Frank B. Hu, Matthias B. Schulze, Clemens Wittenbecher

**Affiliations:** 1 Department of Molecular Epidemiology, German Institute of Human Nutrition Potsdam-Rehbruecke, Nuthetal, Germany; 2 German Center for Diabetes Research (DZD), Neuherberg, Germany; 3 Hugh Sinclair Unit of Human Nutrition, Institute for Cardiovascular and Metabolic Research and Institute for Food, Nutrition and Health, Reading, UK; 4 Human Nutrition Unit, Department of Biochemistry and Biotechnology, Institut d’Investigació Sanitària Pere Virgili, Universitat Rovira i Virgili, Reus, Spain; 5 Consorcio CIBER, M.P. Fisiopatología de la Obesidad y Nutrición (CIBERObn), Instituto de Salud Carlos III (ISCIII), Madrid, Spain; 6 Department of Preventive Medicine and Public Health, IdiSNA (Instituto de Investigación Sanitaria de Navarra), University of Navarra, Pamplona, Spain; 7 Department of Internal Medicine, Institut d’Investigacions Biomèdiques August Pi Sunyer (IDIBAPS), Hospital Clinic, University of Barcelona, Barcelona, Spain; 8 Department of Public Health and Caring Sciences, Clinical Nutrition and Metabolism, Uppsala University, Uppsala, Sweden; 9 Division of Women’s Health, Department of Medicine, Brigham and Women’s Hospital, Boston, MA USA; 10 Department of Public Health and Novo Nordisk Foundation Center for Basic Metabolic Research, Faculty of Health and Medical Sciences, University of Copenhagen, Copenhagen, Denmark; 11 Department of Nutrition, Harvard T.H. Chan School of Public Health, Boston, MA USA; 12 Department of Epidemiology, Harvard T.H. Chan School of Public Health, Boston, MA USA; 13 Channing Division of Network Medicine, Department of Medicine, Brigham and Women’s Hospital and Harvard Medical School, Boston, MA USA; 14 Institute of Nutritional Science, University of Potsdam, Nuthetal, Germany; 15 Department of Life Sciences, SciLifeLab, Chalmers University of Technology, Gothenburg, Sweden

**Keywords:** Predictive markers, Risk factors, Cardiovascular diseases, Endocrine system and metabolic diseases

## Abstract

Current cardiometabolic disease prevention guidelines recommend increasing dietary unsaturated fat intake while reducing saturated fats. Here we use lipidomics data from a randomized controlled dietary intervention trial to construct a multilipid score (MLS), summarizing the effects of replacing saturated fat with unsaturated fat on 45 lipid metabolite concentrations. In the EPIC-Potsdam cohort, a difference in the MLS, reflecting better dietary fat quality, was associated with a significant reduction in the incidence of cardiovascular disease (−32%; 95% confidence interval (95% CI): −21% to −42%) and type 2 diabetes (−26%; 95% CI: −15% to −35%). We built a closely correlated simplified score, reduced MLS (rMLS), and observed that beneficial rMLS changes, suggesting improved dietary fat quality over 10 years, were associated with lower diabetes risk (odds ratio per standard deviation of 0.76; 95% CI: 0.59 to 0.98) in the Nurses’ Health Study. Furthermore, in the PREDIMED trial, an olive oil-rich Mediterranean diet intervention primarily reduced diabetes incidence among participants with unfavorable preintervention rMLS levels, suggestive of disturbed lipid metabolism before intervention. Our findings indicate that the effects of dietary fat quality on the lipidome can contribute to a more precise understanding and possible prediction of the health outcomes of specific dietary fat modifications.

## Abstract (web summary)

Changes in lipidome profiles, as reflected by improvements in dietary fat quality from saturated to unsaturated fats, were associated with reduced cardiometabolic disease risk, and high-risk populations with unhealthy lipidome profiles would most benefit from an olive oil-rich Mediterranean diet.

## Main

Cardiovascular diseases (CVDs) account for approximately 20 million (34%) global deaths annually^1^. In addition, type 2 diabetes (T2D) substantially contributes to global noncommunicable disease burden and premature mortality, primarily through its vascular complications^2^. Therefore, reductions in cardiometabolic disease (that is, CVD and T2D) incidence yield substantial societal benefits^3,4^. The World Health Organization (WHO) recently issued dietary guidelines that advocate for reducing saturated fats while increasing unsaturated fats to prevent cardiometabolic diseases^5^, in line with evidence synthesis efforts and national guidelines that emphasize the importance of the type and quality of fats in the habitual diet^6,7^.

However, current controversies concerning the role of dietary fat in cardiometabolic health center on the potential metabolic benefits of a high-fat, low-carbohydrate diet (LCD) versus the merits of reducing saturated fat intake. For example, dairy products are high in saturated fatty acids (SFAs), and yet observational data indicate that their relation to cardiometabolic risk may be neutral or possibly beneficial, especially when compared to low levels of these foods or foods that are high in refined carbohydrates. The specific effect of replacing SFAs from animal sources with plant-based unsaturated fatty acids (UFAs) in the context of a moderately high-fat diet on cardiometabolic risk, including T2D and CVD, is still unclear. Given the complex, long-term nature of dietary impacts on health, definitive endpoint trials remain elusive.

Some populations appear to be especially vulnerable to the negative health impacts of specific diets^5^. The interplay of genetics, physiological traits and diet influences lipid metabolism and cardiometabolic disease development^8–12^. Therefore, a beneficial dietary fat composition may be especially critical in groups predisposed to dysregulated lipid metabolism.

Clinical lipid markers, like blood lipoproteins and triglycerides, commonly used as surrogates for cardiometabolic disease risk, are affected by dietary fat^13–15^. However, recent evidence challenges the traditional view that dietary fat quality influences cardiometabolic health primarily through these standard blood lipid profiles^16^. More complex effects of dietary fat quality on lipoprotein size and composition, as well as the direct involvement of specific lipid compounds in signal transduction, membrane fluidity and immune response, have been demonstrated^16^. Improved markers of metabolic adaptation to dietary fat quality also create opportunities to integrate data from shorter dietary randomized controlled trials (RCTs) and prospective cohort studies with long follow-ups and substantial numbers of incident disease cases.

Our recent studies have corroborated that comprehensive lipidomics profiles are susceptible to dietary fat modification and are strongly associated with cardiometabolic risk^17–19^. Other observational studies have generated multimetabolite signatures of dietary exposures, including a plant-based diet, dairy intake and a Mediterranean diet, and associated them with disease risk^20–26^. However, assessing diet effects on the metabolome in a well-conducted RCT reduces measurement error, rules out confounding by other lifestyle factors and relates metabolite signatures to a precisely defined diet substitution. Multistudy integration based on overlapping lipidomics profiling data can strengthen the evidence on the long-term health effects of dietary fat quality and offer potential applications in biomarker-driven precision nutrition^27,28^.

Here we test the hypothesis that alterations of the lipidome interlink the replacement of dietary SFAs with UFAs and cardiometabolic disease risk. We integrate lipid profiling data from dietary RCTs, which offer advantages in precise control of dietary exposures and protection against confounding, and large prospective cohort studies with substantial sample sizes, real-life dietary data and long duration of comprehensive phenotyping to examine the lipidome changes that relate dietary fat quality with cardiometabolic risk and examine potential precision nutrition applications.

## Results

### Study design

**Fig. 1:**
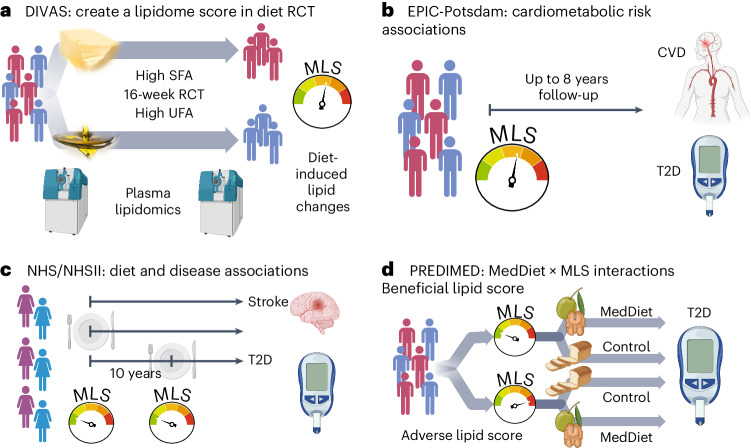
Study design. **a**, DIVAS trial. First, we use the DIVAS trial to generate an MLS of controlled unsaturated-for-saturated fat substitution. **b**, EPIC-Potsdam cohort. Second, we reconstruct the MLS and use it as a surrogate marker to estimate the expected cardiometabolic risk effects of the DIVAS intervention diet. **c**, NHS/NHSII cohorts. Third, we construct a highly correlated rMLS based on another lipidomics platform and link baseline levels and 10-year changes of this rMLS to cardiometabolic disease risk. **d**, PREDIMED trial. Fourth, we examine potential differences in the Mediterranean diet intervention effect on T2D risk across participants with different preintervention rMLS levels (effect modification). Figure created with BioRender.com.

We generated a multilipid score (MLS) based on 45 of 111 analyzed lipid class-specific fatty acid concentrations using preintervention and postintervention lipidomics data in the Dietary Intervention and VAScular function (DIVAS) trial. DIVAS is a 16-week RCT comparing an SFA-rich diet to a diet high in plant-based UFAs^29^. Higher MLS levels reflect the effect of replacing dietary SFAs with plant-based UFAs on the lipidome (Fig. 1a). Using the population-based European Prospective Investigation into Cancer and Nutrition (EPIC)-Potsdam cohort study with the same lipidomics data, we linked the MLS that reflects better dietary fat quality to future cardiometabolic disease risks (Fig. 1b)^30,31^.

We partially validated the DIVAS dietary SFA with UFA replacement effects on circulating lipid metabolites in the LIPOGAIN-2 trial, an 8-week overfeeding RCT with an SFA-enriched diet in the control group and a UFA-enriched diet intervention arm^32^. We also analyzed the Nurses’ Health Study (NHS) and NHSII cohorts and the Prevención con Dieta Mediterránea (PREDIMED) trial with Broad Institute lipidomics data, which provided relative abundances of a subset of the lipid metabolites included in the original MLS. A reduced MLS (rMLS) limited to lipids available on both platforms (42 lower-resolution lipid variables to reflect 15 class-specific fatty acid concentrations) was strongly correlated with the original MLS. In the NHS/NHSII cohorts, we replicated diet and disease associations and also related 10-year increases in rMLS levels (suggesting improved dietary fat quality) with subsequent T2D risk (Fig. 1c). In the PREDIMED trial, we examined if individuals with adverse preintervention rMLS levels, suggestive of unfavorable dietary fat quality before the intervention, benefit more from a Mediterranean diet intervention, which is high in plant-based UFAs, particularly from nuts and olive oil, and has been shown to lower CVD and T2D risk (Fig. 1d)^33,34^. The baseline characteristics of all studies are provided in Supplementary Tables 1–9.

### An intervention-derived lipidomics score

**Fig. 2:**
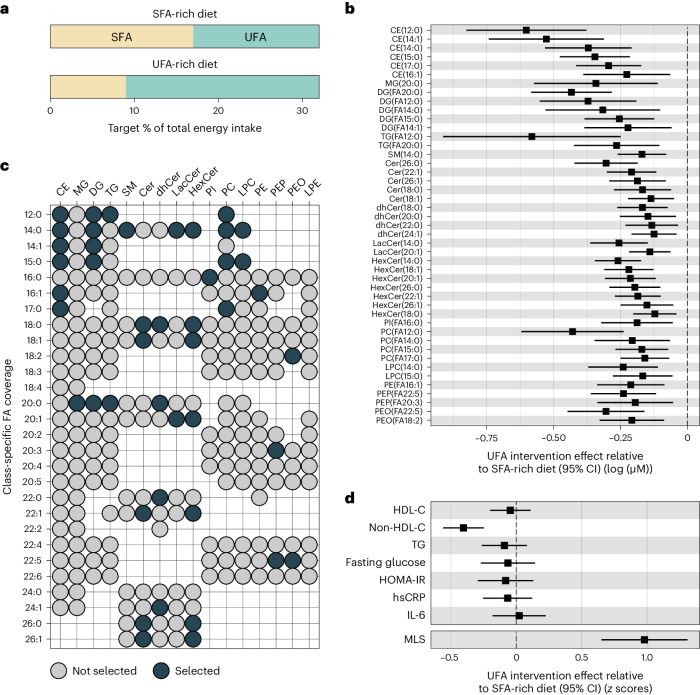
Deriving an MLS of modified dietary fatty acid composition in the DIVAS trial and benchmarking against established risk biomarkers. **a**, Target percentage of total energy intake from SFA and UFA sources per intervention arm. **b**, Effect of UFA-rich diet interventions relative to the SFA-rich diet on lipid concentrations (*n* = 113) modeled as postintervention concentration differences (95% CI) between SFA- and UFA-rich diets adjusting for baseline concentration, age, BMI and sex. Only statistically significantly changed lipids (FDR < 0.05) are shown. The center indicates the β-coefficient, and whiskers indicate 95% CIs. **c**, Selected lipids (FDR < 0.05) for the MLS calculation according to lipid class and fatty acid. **d**, Effect of UFA-rich diet interventions relative to the SFA-rich diet on MLS and established risk biomarkers (*n* = 113). MLS was calculated as weighted sum. Observed intervention effects served as weights. For comparison, MLS and risk biomarkers were variance standardized (unit = 1 s.d.). Data were modeled as postintervention score or concentration differences between SFA- and UFA-rich diets adjusting for baseline score level or concentration, age, BMI and sex. Results for established risk markers were originally published in Vafeiadou et al.^29^. The center indicates the β-coefficient, and the whiskers indicate the 95% CI. FA, fatty acid; TG, triglycerides; HOMA-IR, homeostatic model assessment for insulin resistance; IL-6, interleukin-6; hsCRP, high-sensitivity C-reactive protein. Source data

**Extended Data Fig. 1:**
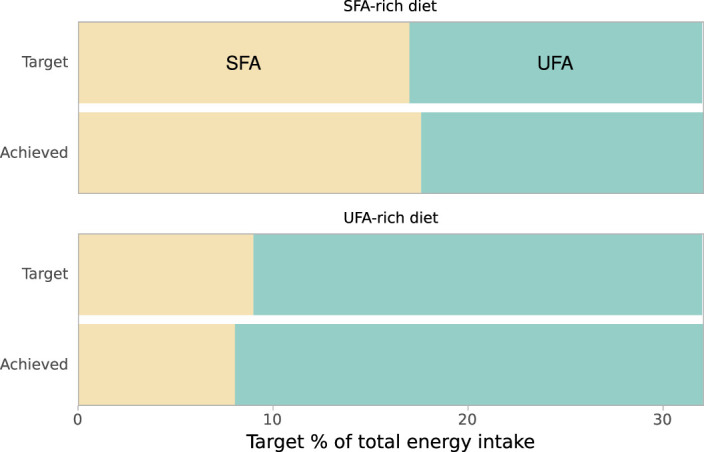
Comparison of target versus average achieved SFA and UFA intake in DIVAS. Achieved SFA and UFA intakes were estimated from 4-day weighed diet diaries collected during the intervention. Source data

We generated a summary score that reflects the influence of dietary fat quality on the lipidome in post hoc lipidomics analyses of the DIVAS dietary intervention trial. In the DIVAS trial, all diets were isoenergetic and provided 36% of the total energy from fats. Nonfat macronutrient intake and omega-3-poly-UFA (PUFA) intake were uniform across all diet groups. The control diet was high in SFAs (SFA-rich diet; 17% total energy from SFAs and 15% total energy from UFAs (11% mono-UFAs (MUFAs) and 4% omega-6-PUFAs); *n* = 65). The DIVAS trial had two intervention arms in which 8% of total energy from SFAs was replaced with 8% of total energy from UFAs, either only with MUFAs (SFA:MUFA:omega-6-PUFA content in percent total energy: 9:19:4) or with a mix of MUFAs and PUFAs (SFA:MUFA:omega-6-PUFA content in percent total energy: 9:13:10). Extensive sensitivity analyses showed that our analysis workflow yielded highly consistent results in the two intervention arms, and we therefore present comparisons between the control group (high SFA intake) and pooled intervention group (high UFA intake). In the pooled intervention group (UFA-rich diet; *n* = 130), dietary targets were 9% of total energy from SFAs and 23% of total energy from UFAs. Detailed dietary assessments yielded an estimated total energy intake contribution of 17.6% by SFAs and 14.5% by UFAs in the SFA-rich diet group and of 8.1% by SFAs and 24% by UFAs in the UFA-rich diet group (Fig. 2a and Extended Data Fig. 1)^29,35^.

A random sample of DIVAS trial participants (*n* = 113; Supplementary Table 2) underwent pre- and postintervention lipidomics profiling, measuring the absolute concentrations of 987 molecular lipid species. We summarized the absolute levels of up to 28 specific fatty acids (12–26 carbon atoms, 0–6 unsaturations) in 16 lipid classes (7 phospholipid (sub)classes, 5 sphingolipid classes, 3 neutral glycerolipid classes and cholesterol esters), generating 111 lipid class-specific fatty acid concentrations. We then compared lipidomics responses to the intervention diet versus to the control diet. After multiple testing correction (false discovery rate (FDR) < 0.05), the replacement of SFAs with UFAs in the diet intervention group significantly reduced the circulating concentrations of 45 class-specific fatty acids (Fig. 2b).

The UFA-rich diet primarily reduced lipid metabolites with medium- or long-chain fatty acid residuals that contain no (for example, C12:0, C14:0, C18:0 and C20:0) or few (for example, C14:1 and C16:1) unsaturations. In descending frequency, the affected lipid metabolites belonged to the classes of ceramides (*n* = 18; including ceramides, dihydroceramides, lactosylceramides and hexosylceramides), cholesterol esters (*n* = 6), phosphatidylcholines (*n* = 6), diglycerides (*n* = 5), phosphatidylethanolamines (*n* = 5; including alkyl- and plasmalogen-phosphatidylethanolamines), triglycerides (*n* = 2), lysophosphatidylcholines (*n* = 2), monoglycerides (*n* = 1), sphingomyelins (*n* = 1) and phosphatidylinositols (*n* = 1; Fig. 2c).

We summarized the statistically significant effects of the UFA-rich diet intervention on lipid metabolites in a weighted MLS. The DIVAS diet effect estimates were defined as score weights. Detailed information to reconstruct the MLS based on absolute lipid concentrations is provided in Supplementary Tables 10 and 11. As such, a higher MLS reflects a higher UFA intake. This MLS increased substantially in the UFA-rich intervention diet group compared to in the SFA-rich diet control group (+0.98 s.d.; Fig. 2d). For comparison, we show the effects of the DIVAS diet interventions on blood lipids (high-density lipoprotein cholesterol (HDL-C), non-HDL-C and triglycerides), glucose markers (fasting glucose and homeostatic model assessment for insulin resistance) and inflammation markers (high-sensitivity C-reactive protein (hsCRP) and interleukin-6). Among these clinical cardiometabolic risk markers, the DIVAS intervention diet only affected non-HDL-C levels (−0.4 s.d., FDR < 0.05; Fig. 2d)^29^.

### Lipidomics score correlations with foods and biomarkers

**Fig. 3:**
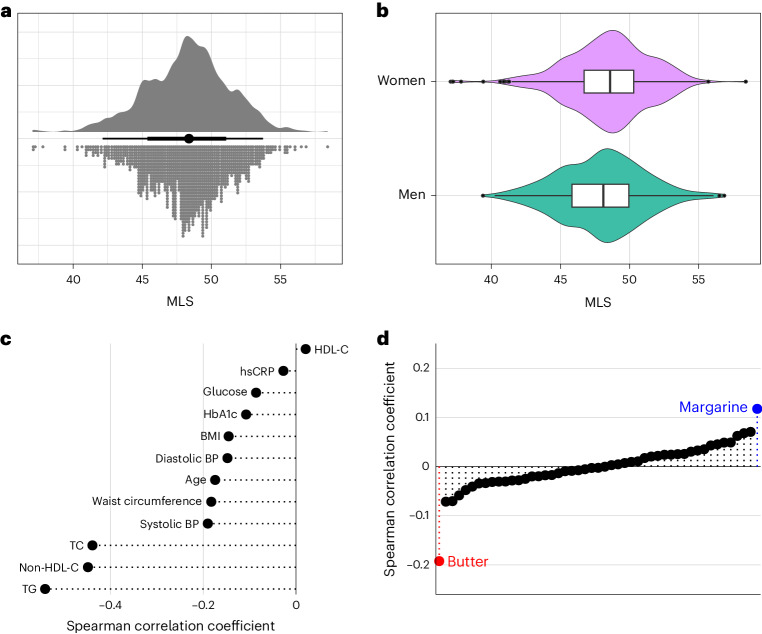
Distribution and correlations of the MLS in the EPIC-Potsdam subcohort. **a**, Univariate distribution of the MLS (*n* = 1,148). **b**, Distribution of the MLS in men (*n* = 438) and women (*n* = 710). The center line indicates the median, hinges indicate the first and third quartiles, and whiskers indicate the median ± 1.5 × interquartile range. **c**, Spearman correlation of the MLS with age, anthropometry, blood pressure and standard clinical biomarkers. **d**, Spearman correlation of the MLS with self-reported habitual intake of different food groups. Highlighted are the strongest positively (margarine) and negatively (butter) correlated foods. BP, blood pressure; TC, total cholesterol. Source data

We constructed the DIVAS-derived MLS in the EPIC-Potsdam cohort using harmonized lipidomics data. This was done within a CVD and T2D case–cohort design. This study included a random subcohort of 1,262 individuals who were representative of the entire cohort without prevalent cardiometabolic conditions. Additionally, we oversampled participants who developed CVD (*n* = 551) or T2D (*n* = 775) during the follow-up period (Supplementary Table 3). The MLS distribution in the subcohort was approximately normal, with similar variance across sexes (Fig. 3a,b).

The MLS weakly inversely correlated with age, body mass index (BMI), waist circumference and blood pressure and moderately inversely correlated with triglycerides, non-HDL-C and total cholesterol (Fig. 3c). Among food groups, the MLS showed the most pronounced positive correlation with margarine and the most pronounced inverse correlation with butter (Fig. 3d). Butter fat contains around 65% SFAs and 28% MUFAs and small amounts of PUFAs and naturally occurring *trans*-fat. Typical margarine contains 80% total fat, of which around 20% are SFAs, 50% are MUFAs and 30% are PUFAs^36^. A comprehensive list of MLS correlations with food group intake in the EPIC-Potsdam cohort is available in Supplementary Table 12. These analyses conducted in a free-living study population show that the MLS correlates in the expected directions with the primary sources of plant-based UFAs and dairy SFAs in participants’ diets.

### Lipidomics score associations with CVD and T2D

In the EPIC-Potsdam cohort, we associated the MLS with cardiometabolic disease risk, standardizing the MLS to the postintervention contrast between the control and intervention groups in the DIVAS trial. Therefore, cardiometabolic risk estimates in the EPIC-Potsdam cohort reflect expected effects of the DIVAS trial diet intervention-induced difference in the MLS. The Cox proportional hazards models were adjusted for age, sex, waist circumference, height, leisure-time physical activity, highest achieved education level, fasting status at blood draw, total energy intake, blood pressure (systolic and diastolic), smoking status, alcohol intake, use of antihypertensive medication, lipid-lowering medication and acetylsalicylic acid medication.

**Fig. 4:**
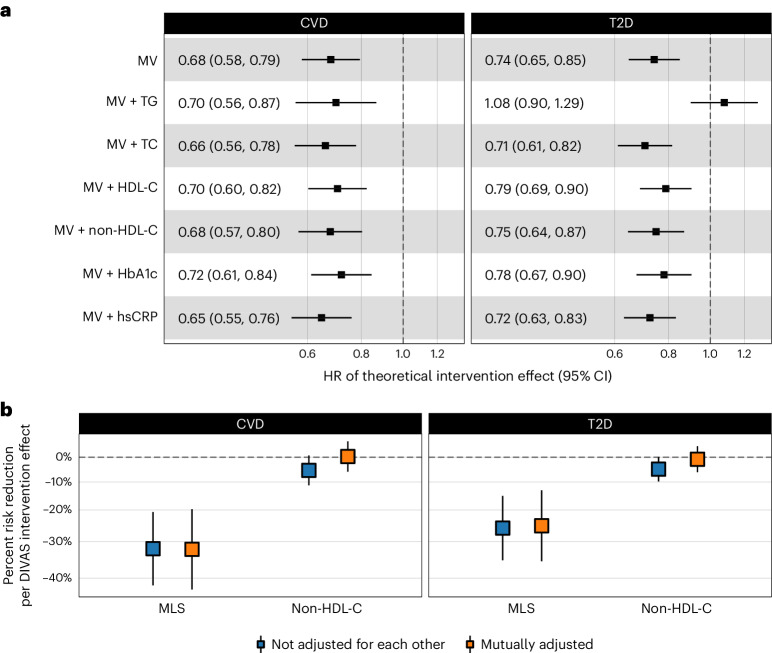
Association of the MLS with CVD and T2D incidence in the EPIC-Potsdam cohort. **a**, MLS–cardiometabolic disease risk associations in different multivariable-adjusted Cox proportional hazards models adjusted for age, sex, waist circumference, height, leisure-time physical activity, highest achieved education level, fasting status at blood draw, total energy intake, blood pressure (systolic and diastolic), smoking status, alcohol intake, use of antihypertensive medication, lipid-lowering medication and acetylsalicylic acid medication (T2D: *n*/cases = 1,886/775; CVD: *n*/cases = 1,671/551). The center indicates the hazard ratio, and the whiskers indicate 95% CI. MV, multivariable adjusted; HR, hazard ratio. **b**, Comparison of multivariable-adjusted disease risk associations between the MLS and non-HDL-C, which was the only significantly affected standard biomarker in the DIVAS trial. The center indicates the percent risk reduction, and whiskers indicate the 95% CI. Source data

In the EPIC-Potsdam cohort, the DIVAS diet-induced MLS difference was associated with 32% (95% confidence interval (95% CI): 21% to 42%) lower CVD (composite endpoint of primary incidence of myocardial infarction (MI) and stroke) and 26% (95% CI: 15% to 35%) lower T2D incidence. Additional adjustment for triglycerides, total cholesterol, HDL-C, non-HDL-C, hemoglobin A1c (HbA_1c_) or hsCRP did not substantially alter the MLS–CVD association. The association of the MLS with T2D risk was rendered statistically nonsignificant after adjustment for triglycerides but was only marginally affected by adjustment for the other standard metabolic risk biomarkers (Fig. 4a).

**Extended Data Fig. 2:**
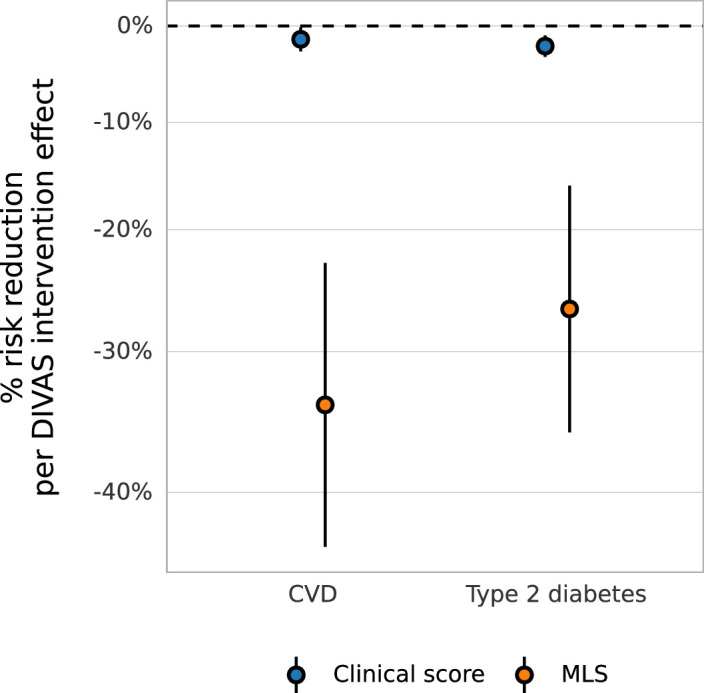
Cardiometabolic risk associations of MLS vs a clinical score based on glucose, HDL-C, non-HDL-C, total triglycerides, and hsCRP. Derivation of clinical score irrespective of prespecified benchmark (FDR < 0.05) used for MLS (n = 113). The clinical score summarizes the nominal effects of the DIVAS diet intervention on HDL-C, non-HDL-C, hsCRP, glucose, and total triglycerides independent of statistical significance. Similar to the MLS, the clinical score was scaled by the observed intervention effect on the clinical score in the DIVAS trial. Source data

The only significant effect of the DIVAS diet on standard surrogate markers was on non-HDL-C. Therefore, we also standardized non-HDL-C on the postintervention contrast between the control and intervention groups in the DIVAS trial. The impact of the DIVAS trial UFA-rich diet intervention on non-HDL-C was associated with a 5% (95% CI: 10% to 0%) lower relative risk of CVD and 5% (95% CI: 11% to 1%) lower relative risk of incident T2D in the EPIC-Potsdam cohort. Thus, the increase in MLS, which reflects improved fat quality, resulted in sixfold stronger CVD (32% versus 5%) and fivefold stronger T2D (26% versus 5%) relative risk reductions compared to the risk reductions associated with changes in non-HDL-C levels (Fig. 4b). When we included the non-HDL-C levels and the MLS in the same mutually adjusted model, the non-HDL-C associations with CVD and T2D risk were attenuated, whereas the MLS associations remained substantial and statistically significant. A sensitivity analysis using a weighted combination of all available established cardiometabolic risk markers (clinical score), independent of the statistical significance of the DIVAS diet intervention effects, yielded similar results. However, the MLS showed stronger inverse disease associations than the clinical score (Extended Data Fig. 2). These results suggest that the MLS may capture the cardiometabolic health impact of altered dietary fat quality in more detail than established surrogate biomarkers.

### Replication of lipidomics score associations with diet

**Extended Data Fig. 3:**
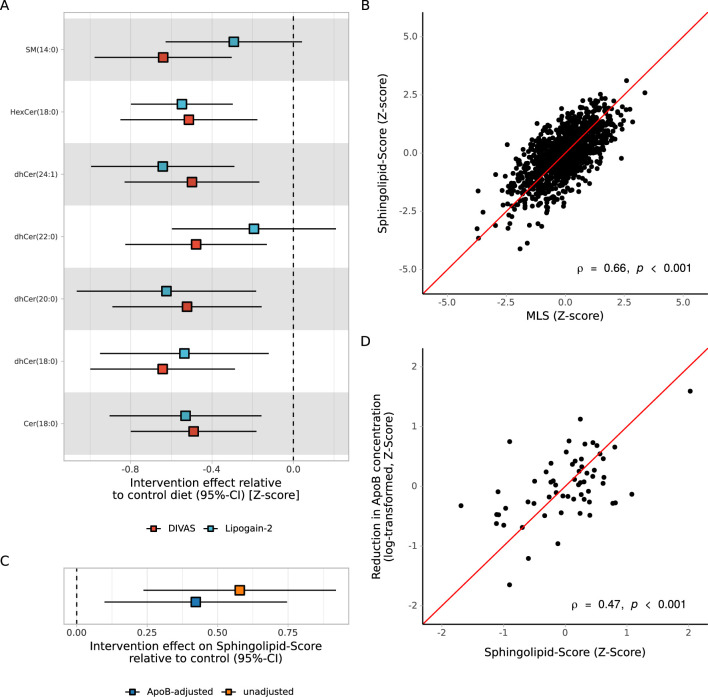
Replication of diet effects on a reduced, sphingolipid-based score (Sphingolipid-Score) in the LIPOGAIN-2 trial. **a**, Comparison of intervention effects on sphingolipids that are part of the MLS and available in the LIPOGAIN-2 trial (n = 60). Center = beta coefficient, whiskers = 95% confidence interval. **b**, Scatter plot and Spearman correlation between MLS and Sphingolipid-Score in the EPIC-Potsdam cohort. Two-sided test, p value < 2.2*10^−16^. **c**, Observed effect of LIPOGAIN-2 intervention on Sphingolipid-Score (n = 60). The Sphingolipid-Score was scaled by the observed effect in the DIVAS trial, therefore a change in one unit indicates the same effect as observed in DIVAS. Center = beta coefficient, whiskers = 95% confidence interval. **d**, Scatter plot and Spearman correlation between change in apolipoprotein B and Sphingolipid-Score in the LIPOGAIN-2 trial. Two-sided test, p-value = 0.00015. Shown values are post-intervention levels adjusted for baseline levels, age, sex, BMI, and intervention. Source data

In post hoc analyses, replacing SFA with UFA overfeeding in the LIPOGAIN-2 trial (*n* = 60; Supplementary Table 4) consistently reduced all seven overlapping sphingolipid concentrations, and the LIPOGAIN-2 trial diet effects on five of these seven sphingolipids were also significant (*P* < 0.05; Extended Data Fig. 3a). We derived a score based on the seven overlapping sphingolipids, which was strongly correlated with the original MLS (Spearman correlation coefficient *ρ* = 0.66; Extended Data Fig. 3b). Diet intervention effects on this sphingolipid score were consistent and similarly significant between the DIVAS and LIPOGAIN-2 RCTs (Extended Data Fig. 3c). The LIPOGAIN-2 diet-induced changes in the sphingolipid score, reflecting lower sphingolipid metabolite concentrations, were moderately correlated with diet-induced reduction in apolipoprotein B count (*ρ* = 0.47; Extended Data Fig. 3d). The LIPOGAIN-2 diet effect on the sphingolipid score was attenuated but remained strong and significant after additional adjustment for apolipoprotein B changes (Extended Data Fig. 3c).

**Extended Data Fig. 4:**
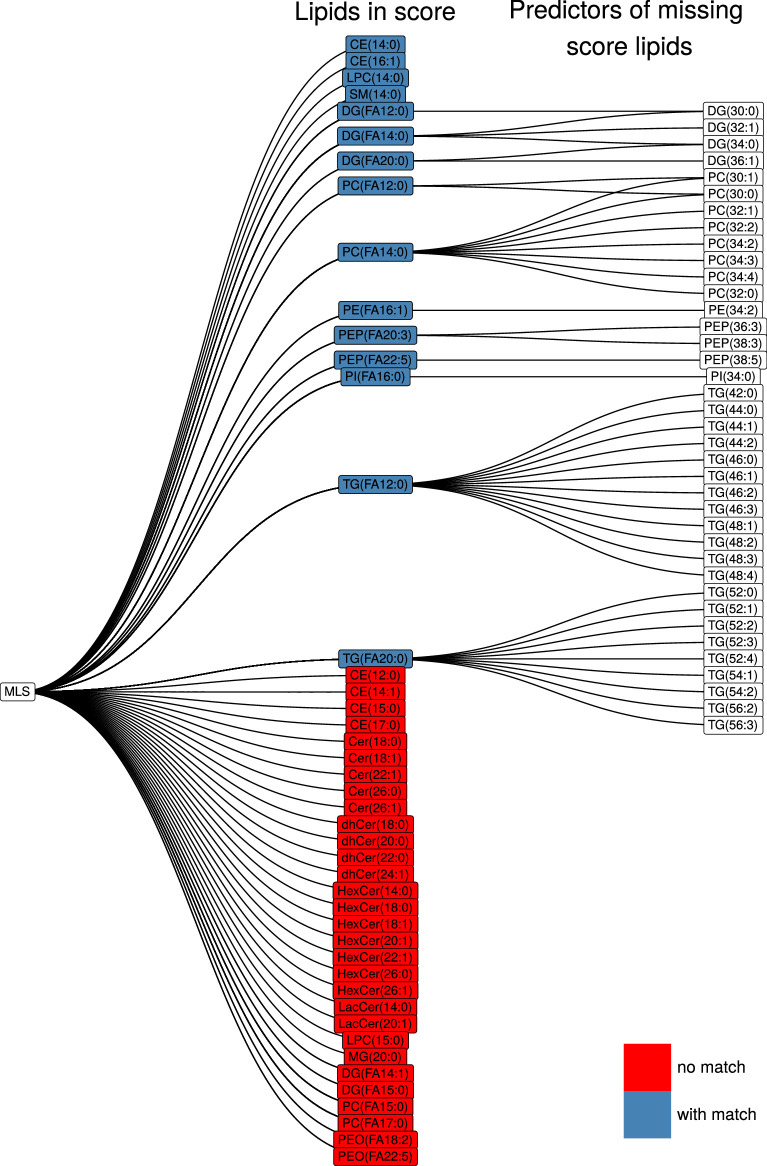
Matching of Metabolon and Broad Institute lipidomics data. Shown are all lipids that are part of the MLS. Color coding indicates presence or absence in rMLS. In case of no direct match between datasets, where possible we generated predicted concentrations based on available isobaric species level in the EPIC-Potsdam cohort.

**Extended Data Fig. 5:**
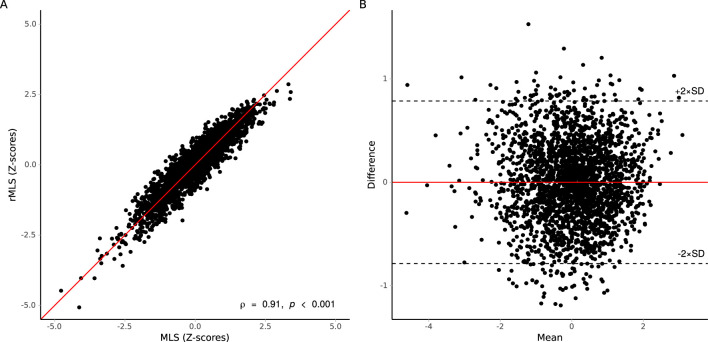
Correlation and agreement of MLS and rMLS. **a**, Scatter plot of the Spearman correlation between MLS based on Metabolon lipidomics data and rMLS based on lipids that are also available through the Broad Insitute lipidomics platform in the EPIC-Potsdam subcohort (n=1,262). Shown is Spearman correlation, two-sided test, p-value < 2.2*10^−16^. **b**, Bland-Altman plot showing the agreement between MLS and rMLS in the EPIC-Potsdam cohort. Source data

A subsample of 10,894 women in the NHS (*n* = 7,479) and NHSII (*n* = 3,412) cohorts have Broad Institute lipidomics data with species-level information on the total number of C atoms and double bonds in the fatty acid residuals of lipid metabolites (Supplementary Tables 5–8)^37^. We derived the rMLS using 42 lower-resolution lipid variables to reflect 15 class-specific fatty acid concentrations in the original MLS (Extended Data Fig. 4). In the EPIC-Potsdam cohort, the rMLS showed high correlation (*ρ* = 0.91; Extended Data Fig. 5a), strong agreement (Extended Data Fig. 5b) and CVD and T2D associations comparable with the original MLS.

**Fig. 5:**
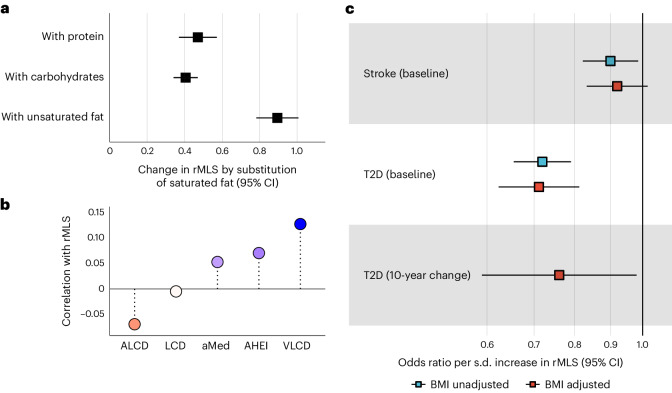
Diet and disease association of the rMLS in the NHS cohorts. **a**, Change in rMLS by substitution of 8% total energy from saturated fat for isocaloric energy intake from alternative macronutrient sources, that is, protein, carbohydrates or unsaturated fats (*n* = 10,381). The center indicates the β-coefficient, and whiskers indicate the 95% CI. **b**, Spearman correlation between established diet scores and rMLS (sample sizes for respective correlations: LCD, *n* = 6,045; animal-based LCD score (ALCD), *n* = 6,045; vegetable-based LCD score (VLCD), *n* = 6,045; aMed, *n* = 6,593; AHEI, *n* = 6,370). **c**, T2D (*n* case–control pairs = 728) and stroke (*n* case–control pairs = 336) risk in relation to baseline rMLS levels and 10-year rMLS change (*n* case–control pairs = 244). Data for change analyses are not available for stroke. The T2D case–control study was matched for age, race, fasting status (all fasted), ethnicity and season of blood collection, and conditional logistic regression models were adjusted for the AHEI, smoking status and subsequently BMI; the stroke case–control study was matched for age, fasting and smoking status, and conditional logistic regression models were adjusted for the AHEI and subsequently BMI. Source data

**Extended Data Fig. 6:**
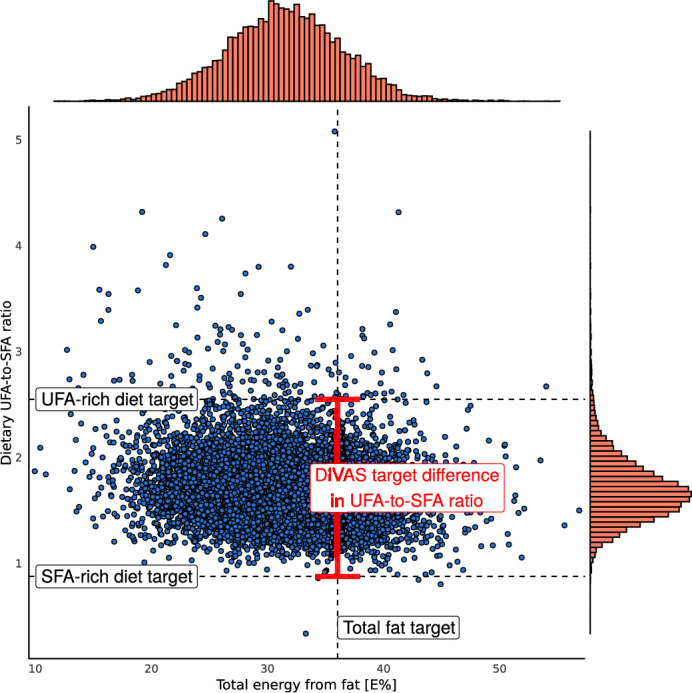
Projection of DIVAS targets for dietary UFA-to-SFA ratio and total energy from fats onto the pooled NHS/NHSII study population. Shown are total energy from fat and dietary UFA-to-SFA ratio of NHS/NHSII cohort participants with metabolomics data (n = 10,381). Source data

**Extended Data Fig. 7:**
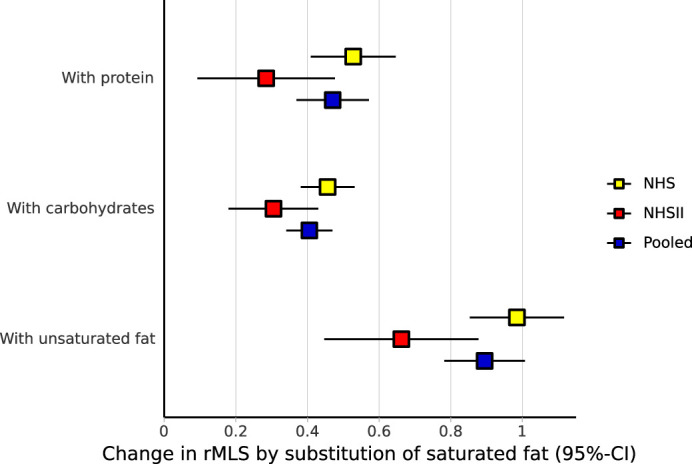
Effect of substituting dietary SFA with other macronutrients on rMLS. Change in rMLS by substitution of 8% total energy from saturated fat for isocaloric energy intake from alternative macronutrient sources, that is, protein, carbohydrates, or unsaturated fats in the Nurses’ Health Study (NHS, n = 7,457) and Nurses’ Health Study II (NHS2, n = 3,412) and the pooled analysis of both cohorts (n = 10,869). Center = beta coefficient, whiskers = 95% confidence interval. Source data

In the NHS/NHSII cohorts, we used the average of the two food frequency questionnaires (FFQs) closest to the blood sample collection to estimate the macronutrient composition of individual diets. A pooled cross-sectional analysis in 9,309 women with complete macronutrient intake data yielded an estimated increase of the rMLS by 0.89 s.d. (*P* = 6.7 × 10^–54^) when modeling the replacement of 8% total energy of dietary SFAs with UFAs (Fig. 5a), a contrast covered by the range of SFA and UFA intake (Extended Data Fig. 6). The replacement of SFAs with other macronutrients (carbohydrates and protein) was associated with a significant but less pronounced increase in the rMLS (Fig. 5a and Extended Data Fig. 7). We also correlated the rMLS with established diet quality scores (Methods). An LCD score reflecting high intake of fat and protein and low intake of carbohydrates was not related to rMLS levels. However, an animal-based LCD score reflecting high intake of animal fat and animal protein was correlated with lower rMLS levels, whereas a vegetable-based LCD score based on high intake of vegetable protein and vegetable fat was correlated with higher rMLS levels. The Alternate Healthy Eating Index (AHEI) and the Alternate Mediterranean Diet Score (aMed) include high intake of unsaturated plant fats as one among several diet quality metrics and were both related to higher rMLS levels, although the positive correlation was less pronounced than the high plant fat intake-focused vegetable-based LCD score (Fig. 5b). These results indicate that reduced SFA intake and higher intake of plant-based UFAs are major determinants of rMLS plasma levels in a human population under natural conditions.

### Replication of lipidomics score associations with diseases

The NHS/NHSII lipidomics sample included a nested 1:1-matched stroke case–control study (*n* = 1,094 women; Supplementary Table 6). Higher rMLS levels, indicative of better dietary fat quality, were associated with a 10% lower relative stroke risk (odds ratio per s.d. = 0.90, 95% CI: 0.82 to 0.98, *P* < 0.05) in age-adjusted models. Further adjustment for BMI and diet quality (AHEI without alcohol points) slightly attenuated this association and rendered it statistically nonsignificant (odds ratio per s.d. = 0.92, 95% CI: 0.83 to 1.02, *P* > 0.05; *n* = 936 after exclusions due to missing covariable information; Fig. 5c). These estimates are directionally consistent with the 18% lower relative stroke risk per standard deviation higher rMLS (*P* < 0.05) that we estimated in a secondary analysis in the EPIC-Potsdam cohort (data not shown).

Lipidomics data were also available in a nested 1:1-matched T2D case–control study in the NHS (*n* = 1,456 women; Supplementary Table 7). In age-matched models, 1 s.d. higher rMLS was associated with 28% lower relative T2D risk (odds ratio per s.d. = 0.72, 95% CI: 0.65 to 0.79, *P* = 6.4 × 10^–12^). Further adjustment for BMI, diet quality and smoking status did not appreciably affect this association of beneficial rMLS levels with lower T2D risk (odds ratio per s.d. = 0.71, 95% CI: 0.62 to 0.81, *n* = 1,114 after exclusions due to missing covariable information; Fig. 5c), which is consistent with the risk estimates in the EPIC-Potsdam cohort.

In addition, a diabetes case–control study (244 cases and 244 controls; Supplementary Table 8) nested in the NHS cohort had repeated lipidomics profiles 10 years apart but before any diabetes cases occurred and complete information on diet, smoking and BMI at both time points. An increase of the rMLS over 10 years suggestive of better dietary fat quality was associated with 24% lower relative risk of subsequent T2D incidence (odds ratio per s.d. = 0.76, 95% CI: 0.59 to 0.98), adjusted for baseline rMLS levels, BMI, age, diet quality and smoking status (Fig. 5c). Additional adjustment for concurrent changes in BMI and diet quality marginally attenuated the association of rMLS changes with subsequent T2D risk (data not shown). These results indicate that rMLS changes over time translate into altered T2D risk and suggest potential MLS applications to monitor diet effects on T2D risk over time.

### The lipidomics score and Mediterranean diet intervention

**Fig. 6:**
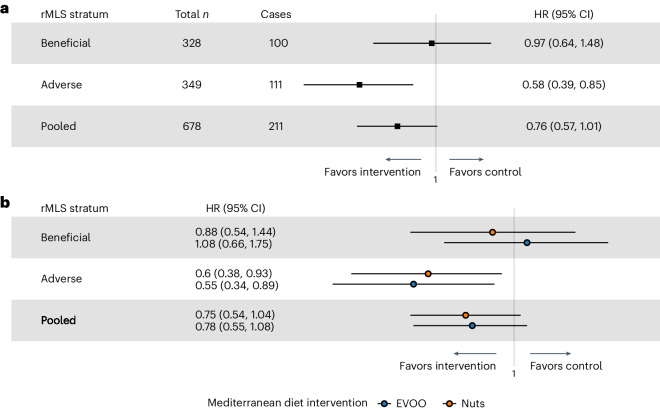
Modification of the effect of Mediterranean diet intervention on T2D by rMLS levels in the PREDIMED trial. **a**, Effect of the Mediterranean diet intervention (pooled nuts and extra virgin olive oil-rich arms) versus a control diet on T2D risk in the PREDIMED trial across preintervention rMLS strata (*n* = 687). **b**, Comparison of the effects of a tree nuts-rich Mediterranean diet intervention and an extra virgin olive oil-rich Mediterranean diet intervention versus a control diet on T2D risk across preintervention rMLS strata. The center indicates the hazard ratio, and whiskers indicate the 95% CI. EVOO, extra virgin olive oil. Source data

The PREDIMED trial showed that a Mediterranean diet reduces CVD and T2D incidence (Supplementary Table 9)^33,34^. Our post hoc analyses revealed a statistically significant interaction between the preintervention rMLS and the Mediterranean diet effect on T2D risk (*P* < 0.05, *n* = 678). Therefore, we examined the Mediterranean diet effect on T2D risk stratified by preintervention rMLS. Participants with lower preintervention rMLS (suggestive of disturbed lipid metabolism and adverse dietary fat quality) showed a 42% (95% CI: 15% to 61%, *n* = 349) reduction in T2D risk by the Mediterranean diet intervention, whereas those with beneficial rMLS levels (above the median) did not show a diabetes risk reduction (reduction in T2D risk for intervention versus control = 3% (95% CI: 36%-decrease to 48%-increase)), *n* = 328; Fig. 6a). These results suggest that adverse levels of the diet-responsive MLS may help to identify population groups with the most robust T2D risk reduction by a Mediterranean diet, possibly informing targeted dietary precision prevention.

Secondary analyses suggested that both Mediterranean diet intervention subtypes with the highest extra virgin olive oil intake and with added nuts were effective in mitigating the adverse preintervention rMLS-related T2D risk (Fig. 6b). Importantly, these analyses are restricted to T2D risk. Participants with a beneficial rMLS level may still benefit from a Mediterranean diet through nonlipid-mediated links with other diseases.

We did not observe a modification of the Mediterranean diet effect on CVD risk by preintervention rMLS (*n* = 736). However, the PREDIMED trial participants were selectively recruited from a high-risk population, and important effects of the Mediterranean diet on CVD risk are likely independent of the dietary fat component, for example, mediated by high polyphenol intake. Further examination of potential interactions between dietary fat quality, the lipidomics-based MLS and long-term CVD risk in other study populations is warranted.

### Network analysis of the diet-related lipidome

**Extended Data Fig. 8:**
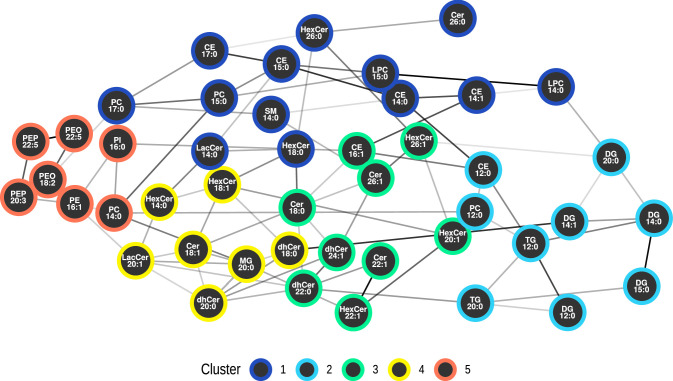
Conditional independence network and clusters of all MLS lipids. The network was derived using the PC-algorithm in the EPIC-Potsdam subcohort (n = 1,262) based on the lipids in the MLS. The nodes in this network correspond to the UFA-rich diet-affected lipids, and links correspond to correlations between a lipid pair that are robust against adjustment for any subset of other network lipids. Many links in this data-driven network connect lipid metabolites that contain the same fatty acid residual but belong to different lipid classes (for example, C12:0 in cholesteryl esters, phosphatidylcholines, triglycerides, and diglycerides; C18:0 in ceramides and hexosylceramides) or lipids of the same class with metabolically related fatty acid residuals (for example, C20:1 and C22:1 hexosylceramides). Based on this network, we used the Louvain modularity detection algorithm to derive data-driven lipid clusters. Cluster 1 was enriched in odd- and short-chain acyl chain-containing cholesterol esters and phospholipids. Cluster 2 mainly featured di- and triglycerides. Cluster 3 was primarily composed of medium to long-chain fatty acid-containing ceramides (including dihydro-, lactosyl-, and hexosyl-ceramides). Cluster 4 was primarily composed of medium to long-chain fatty acid-containing ceramides (including dihydro-, lactosyl-, and hexosyl-ceramides). Cluster 5 was dominated by phosphatidylethanolamines (including those with ether bonds). Abbreviations – MLS: multi-lipid score; HR, hazard ratio; UFA: unsaturated fatty acids; CVD: cardiovascular disease; T2D: type 2 diabetes. Source data

**Extended Data Fig. 9:**
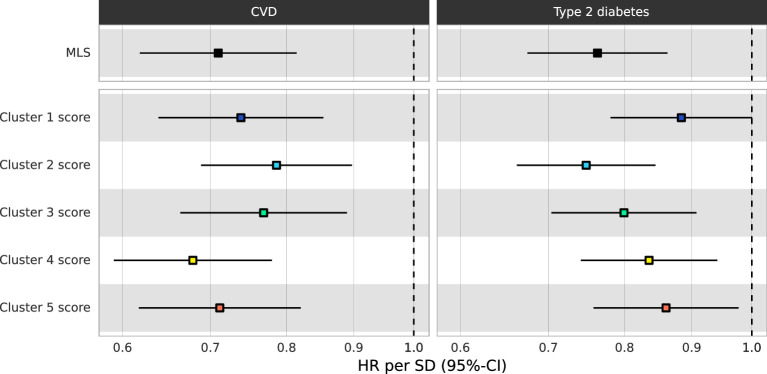
MLS cluster-specific risk associations in the EPIC-Potsdam cohort. Multi-variable-adjusted Cox proportional hazards models adjusted for age, sex, waist circumference, height, leisure-time physical activity, highest achieved education level, fasting status at blood draw, total energy intake, blood pressure (systolic and diastolic), smoking status, alcohol intake, use of anti-hypertensive medication, lipid-lowering medication, and acetylsalicylic acid medication. The CVD risk associations were remarkably robust when restricted MLS were constructed based only on lipids from each cluster. The strongest CVD risk association was assessed for the Cluster 4-based MLS, and all cluster-restricted MLS were associated with a substantial and statistically significant CVD risk reduction. The T2D risk associations of the cluster-restricted MLS were also all directionally consistent with a T2D risk reduction related to the UFA-rich diet imprint. The di- and triglyceride-dominated cluster 2 was particularly informative for T2D risk. The T2D risk association of the hexosyl- and lactosyl-ceramide-enriched cluster 1-based MLS was suggestively inverse but not statistically significant. These robust inverse cardiometabolic risk associations of UFA-rich diet imprint in granular lipidomics network clusters suggest that the depth of lipidomics profiling may be more critical than breadth. However, it appears that ceramide and phosphatidylethanolamine metabolites are particularly informative for the link between UFA-rich diets and CVD risk. At the same time, reduced concentrations of medium and long-chain fatty acid-containing di- and triglycerides are critical to linking UFA-rich diets to T2D risk. Center = hazard ratio, whiskers = 95% confidence interval. Abbreviations – MLS: multi-lipid score; HR, hazard ratio; UFA: unsaturated fatty acids; CVD: cardiovascular disease; T2D: type 2 diabetes. Source data

We derived a conditional independence network in the EPIC-Potsdam subcohort, including all lipid metabolites in the MLS. This data-driven network tended to connect lipid metabolites with biological similarities (that is, containing the same fatty acid residuals or belonging to the same lipid class; Extended Data Fig. 8). We used the Louvain modularity detection algorithm to derive data-driven lipid clusters^38^. We then calculated cluster-specific lipid scores. All cluster-specific scores were associated with a substantial and statistically significant CVD risk reduction. A di- and triglyceride-dominated cluster was particularly informative for T2D risk, whereas the T2D risk association of a cluster enriched in odd- and short-chain acyl chain-containing cholesterol esters and phospholipids was not statistically significant (Extended Data Fig. 9).

**Extended Data Fig. 10:**
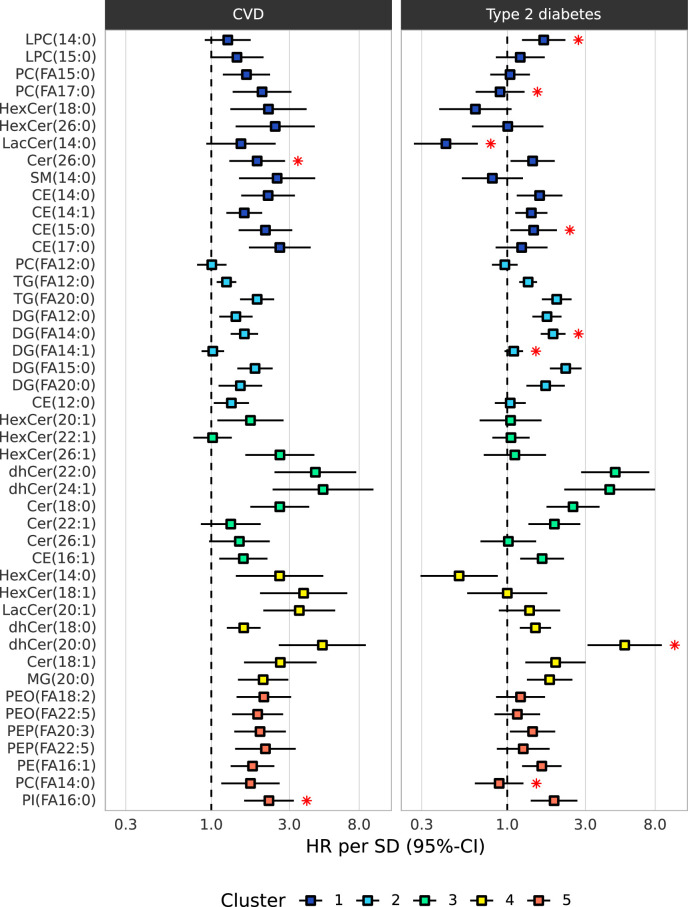
Associations of MLS-contained lipids with cardiometabolic risk in the EPIC-Potsdam cohort. Associations are derived from multi-variable-adjusted Cox proportional hazards models adjusted for age, sex, waist circumference, height, leisure-time physical activity, highest achieved education level, fasting status at blood draw, total energy intake, blood pressure (systolic and diastolic), smoking status, alcohol intake, use of anti-hypertensive medication, lipid-lowering medication, and acetylsalicylic acid medication. Red asterisks indicate that these lipid-disease associations were robust against adjustment for interrelated diet-affected lipids. Center = hazard ratio, whiskers = 95% confidence interval. Abbreviations – HR: hazard ratio; SD: standard deviation; CVD: cardiovascular disease; T2D, type 2 diabetes. Source data

Our analysis revealed that lipid metabolites reduced by the DIVAS trial high-UFA diet and included in the MLS were mostly neutral or associated with high cardiometabolic risk (Extended Data Fig. 10). We used the NetCoupler algorithm to identify direct links between metabolites and disease risk that cannot be explained by adjustment for other metabolites^39,40^. Lipid metabolites with endpoint associations that remain significant after adjustment for other MLS lipids are highlighted in Extended Data Fig. 10, which may help to identify the biological processes that drive the MLS to cardiometabolic risk associations.

## Discussion

In this study, we showed that lipidomics-based MLSs, reflecting the isocaloric replacement of SFAs with UFAs, were associated with significantly lower CVD and T2D risk. The primary analysis of the same lipidomics data in the DIVAS trial and the EPIC-Potsdam cohort yielded more substantial extrapolated cardiometabolic risk reductions than standard cardiometabolic risk markers. Additionally, we demonstrate consistent associations between lipid metabolites and diet and cardiometabolic risk across different cohorts and trials using partially overlapping lipidomics data and show that changes in dietary fat quality-related lipid scores over 10 years precede changes in T2D risk. Post hoc analyses of the PREDIMED trial suggest that adverse preintervention levels of a fat quality-related lipid score may predict a stronger T2D risk reduction by the healthy Mediterranean diet intervention.

Seminal precision nutrition studies have focused on acute metabolic responses to tailor personalized diets^41–43^. This approach can be complemented by biomarker-driven stratified nutrition^44^. Our study supports the concept of using lipidomics multimarker approaches to assess and predict the impact of dietary fat quality on health and improve precision in dietary risk prevention using triangulation of different study designs. We demonstrate the effect of dietary fat quality on the lipidome in a controled RCT with high compliance, which substantially reduces the risk of bias and enhances precision of the dietary exposure compared to observational data. We then leverage large cohort studies to show the relationship between intervention-responsive lipid scores and real-world dietary behaviors and long-term cardiometabolic health and use a lipid score to predict subgroup effects of dietary changes in post hoc analyses of a hard endpoint trial.

The WHO recommends that adults reduce SFA intake to 10% of total energy^5^. Additionally, the WHO guidelines on dietary fat suggest further reducing saturated fat intake below 10% and replacing it with PUFAs and MUFAs from plant sources or fiber-rich carbohydrates^5^. These recommendations rely on a comprehensive synthesis of evidence from trials and observational studies linking lower SFA intake to reduced mortality and CVD risk^15,45,46^. Despite data from over 56,000 trial participants and approximately 3.7 million observational study participants, there is considerable heterogeneity in total fat and SFA intake levels, nutrients and food sources replacing dietary SFAs and duration in the underlying studies and limited data from trials on hard endpoints^5^. Additionally, the evidence of the effect of dietary fat quality on other endpoints, such as T2D^47^, is not reflected in these guidelines. Therefore, the certainty of evidence for these WHO recommendations ranges from very low to moderate, subjecting the guidance on dietary fat quality to ongoing controversies.

In our study, we relate the lipidomics signature of replacing a high-dairy, SFA-rich diet with a diet rich in plant-based MUFAs and PUFAs to the risk of developing CVD and T2D. Our findings from the lipidomics-based integrated analysis of trial and cohort data on CVD risk are consistent with the current recommendation to replace dietary SFAs with MUFAs and PUFAs from plant sources, relating a specific dietary strategy consistent with the DIVAS intervention to quantitative CVD and T2D risk reduction estimates. Additionally, our results show that changes in dietary fat quality-related lipidomics scores over 10 years are significantly associated with a reduction in subsequent T2D risk. Very few studies associate longitudinal lipidomics data with subsequent disease risk^48,49^.

Several studies suggest that higher levels of dairy intake biomarkers are related to reduced T2D risk, although questions concerning potential confounding factors, such as sex, lipid class interactions and differentiation among dairy product types, require further exploration^50,51^. In addition, multimetabolite signatures derived from self-reported dairy consumption showed inverse diabetes associations^22,23^. These metabolite signatures are tuned to distinguish dairy intake from the average diet and may underrepresent lipid metabolites with medium- and long-even-chain SFAs, which are predominant in full-fat dairy but are also abundant in the broader diet. Therefore, it remains unclear to what extent the inverse T2D associations of dairy intake-related metabolite signatures are attributable to dairy fat, other dairy components or correlated factors, such as total dietary fat content. In our study, a lipid score that reflects the metabolic adaptation to the controled replacement of saturated dairy fat with plant-based unsaturated fat was associated with significantly reduced T2D risk. Although our results do not dispute the potential health benefits of total or specific dairy products within a balanced diet^52^, they imply that replacing SFAs from dairy with high-quality plant-based UFAs in a moderately high-fat diet may confer additional cardiometabolic benefits.

Most established biomarker adjustments did not affect MLS–disease associations, except for substantial attenuation of the MLS–T2D association by adjustment for total triglyceride levels. However, the DIVAS diet intervention did not significantly affect triglyceride levels, which is consistent with similar dietary RCTs^53^. Insulin resistance affects very-low-density lipoprotein kinetics and plasma triglyceride levels^54^, but longer interventions might be needed to show these indirect effects. For example, long-term supplementation of high-dose long-chain PUFAs has beneficial effects on triglyceride levels, and replacement of dietary SFAs with UFAs decreases hepatic insulin resistance and liver triglycerides^55–58^. The potential etiological relationship between dietary fatty acid composition, the lipidome, insulin resistance and circulating trigylcerides warrants further investigation.

The Mediterranean diet’s comprehensive health and environmental benefits justify its recommendation for the general population^59^. The Mediterranean diet can reduce T2D incidence, partially due to its high olive oil and low SFA content^34,60,61^. Previous interaction analyses did not detect an effect modification between dietary fat quality and genetic background on T2D risk^62^. We conducted effect modification analysis, showing that the Mediterranean diet-related reduction of T2D incidence in the PREDIMED trial was primarily observed in individuals with adverse preintervention rMLS levels. These observations suggest that the beneficial effects of the Mediterranean diet’s favorable fat quality on T2D risk are particularly pronounced in individuals with disturbed lipid metabolism and unfavorable dietary fat quality, as reflected in adverse preintervention rMLS levels. Therefore, our findings support the concept that lipidomics-based scores may help identify vulnerable population groups and more precisely target dietary interventions focusing on fat quality for T2D prevention.

A strength of this study is the integration of dietary interventions and nutritional cohort studies through lipidomics to examine the impact of dietary fat quality on cardiometabolic risk. We included all significantly DIVAS diet-affected lipids in the MLS, and our network and cluster analyses suggest that the included lipids provide partially redundant information. Including a broad panel of lipids in the primary MLS allowed us to project our findings onto studies with other partially overlapping lipidomics data (LIPOGAIN-2, NHS/NHSII and PREDIMED) and may offer flexibility to consider technical and economic benchmarks for creating targeted precision nutrition biomarker panels. Specificity is a critical concern for dietary intake biomarkers, but this is not applicable here given our study’s focus on MLSs for monitoring the metabolic adaptations to changes in dietary fat quality and inferring the potential impact on cardiometabolic risk. We show that healthful dietary patterns that include, but are not limited to, a high intake of plant-based UFAs correlate with beneficial lipid scores, whereas higher adherence to an animal fat-rich dietary pattern correlates with adverse lipid scores. Other health-related dietary exposures may have convergent effects on the metabolic processes reflected in dietary fat quality-responsive MLSs.

However, this study has several limitations. We did not conduct independent intervention studies to validate absolute effect sizes on all metabolites, establish thresholds or assess cost-effectiveness, which are important steps toward potential future biomarker applications. Applying lipidomics scores to guide dietary risk prevention needs to be tested in de novo RCTs. Additionally, as our study populations were primarily of European ancestry, validating lipid score associations in populations not of European ancestry is essential. The DIVAS trial offered a comprehensive panel of established surrogate biomarkers, but more detailed lipoprotein measurements were not available. The LIPOGAIN-2 correlation analyses of dietary fat quality-induced changes in sphingolipid score and apolipoprotein B count suggest partially overlapping and partially independent diet effects. Further studies into the interplay between dietary fat quality, lipidomics profiles, detailed lipoprotein subclass assessment and prospective disease risk are warranted.

Our analysis focused on the DIVAS trial intervention diet, replacing specific saturated fats (primarily medium- and long-chain SFAs from dairy) with unsaturated fats (mainly plant-based oils and nuts). Similar studies on complementary dietary exposures may inform alternative dietary strategies to produce similar lipidome changes or help compile broader panels of metabolic adaptation markers. In addition, outcome-optimized lipidomics scores have been developed for disease risk prediction^63,64^, and evaluation of the overlap with the dietary fat quality-related alterations in the lipidome may foster progress toward robust and reliable lipidomics-based biomarkers of diet and disease risk.

In conclusion, we selected lipid metabolites that are affected by replacing dietary SFAs with plant-based UFAs in RCTs and showed that scores derived from these lipids are associated with self-reported dietary fat sources, dietary fatty acid composition and health-related dietary pattern adherence in free-living individuals. Lipidomics scores that reflect lower SFA intake and high plant-based UFA intake were consistently associated with reduced incidence of T2D and CVD in prospective cohort studies. The associations of lipidomics scores with diet and disease risk are stronger than established surrogate markers, yielding larger estimated cardiometabolic benefits of improved dietary fat quality. Our findings corroborate the cardiometabolic benefits of replacing dietary SFAs with plant-based UFAs by integrating data from RCTs and nutritional cohorts and suggest that lipidomics-based scores may provide sensitive metrics for the health-related metabolic adaptation to change in dietary fat quality.

## Methods

### Study designs and populations

#### DIVAS trial

Lipidomics analysis was performed in a subset of participants (*n* = 113 of 195) from the DIVAS trial, a 16-week, single-blind randomized controled parallel trial (registered at www.clinicaltrials.gov under accession number NCT01478958). The DIVAS trial was conducted according to the guidelines of the Declaration of Helsinki, and favorable ethical opinion for conduct was given by the West Berkshire Local Research Ethics Committee (09/H0505/56) and the University of Reading Research Ethics Committee (09/40). All individuals provided written informed consent before participating. This study recruited men and women aged between 21 and 60 years and with estimated moderate CVD risk who were randomized to one of three isoenergetic diets: rich in SFAs, rich in MUFAs or rich in mixed UFAs including both MUFAs and omega-6 PUFAs. The target compositions (percent total energy of total fat:SFA:MUFA:PUFA) were 36:17:11:4 for the SFA-rich diet (*n* = 38), 36:9:19:4 for the MUFA-rich diet (*n* = 39) and 36:9:13:10 for the mixed UFA-rich diet (*n* = 36). We collapsed the MUFA-rich and mixed UFA-rich diets into one UFA-rich diet arm for the generation of the MLS.

In the DIVAS dietary intervention trial, all participants’ diets were isoenergetic and provided 36% of total energy (percent total energy) from fats. Nonfat macronutrient intake and sources were consistent between the intervention and control diets. However, different spreads, oils, dairy products and snacks were used to modify the diets’ SFA:UFA ratio. The control diet was high in saturated fat (SFA-rich diet; 17% of total energy from SFAs and 15% of total energy from UFAs; *n* = 38 with lipidomics data). In the intervention diet, 8% of total energy from SFAs was substituted for 8% of total energy from UFAs (UFA-rich diet; 9% of total energy from SFAs and 23% of total energy from UFAs; *n* = 75 with lipidomics data). The analysis of 4-day weighed diet diaries indicated successful implementation of these dietary targets over the intervention period (Fig. 2a)^29,35^. The SFA:MUFA:omega-6-PUFA content in percent total energy in the control group was 17:11:4 and was either 9:19:4 or 9:13:10 in the intervention group arms with different MUFA:PUFA ratios. The omega-3-PUFA content was standardized across all diet groups. Extensive sensitivity analyses revealed that our analysis workflow yielded highly consistent results in the two intervention arms. Therefore, we present comparisons between the control group (high SFA intake) and a pooled intervention group (high UFA intake). We collapsed the MUFA-rich and mixed UFA-rich diet into one UFA-rich diet arm to generate the MLS.

All participants were nonsmokers; were not pregnant or lactating; had normal blood biochemistry and liver and kidney function; did not take dietary supplements or medication for hypertension, raised lipids or inflammatory disorders; had no prior diagnosis of MI, stroke or diabetes; did not consume excessive amounts of alcohol (males: less than 21 U per week; females: less than 14 U per week) and performed fewer than three 30-min sessions of aerobic exercise per week. The trial was single blinded, and randomization was conducted by a study researcher using minimization stratified for sex, age, BMI and estimated CVD risk. The participants were unaware of the assigned intervention diet and were asked to replace habitually consumed sources of exchangeable fats with study foods (spreads, oils, dairy products and commercially available snacks) of specific fatty acid composition provided free of charge.

Dietary guidance was provided at baseline and throughout the study via 1:1 verbal and written instructions. Compliance was monitored through weighed 4-day diet diaries (weeks 0, 8 and 16), records of study food intake and plasma phospholipid fatty acids as short-term biomarkers of intake (weeks 0 and 16). Observed fatty acid intake compositions were largely in line with the defined target fatty acid compositions^35^. Body weight, which was to remain constant, was monitored every 4 weeks, and changes were addressed with advice to the participants to adapt study food or carbohydrate consumption and/or activity levels. Fasting blood samples were taken at baseline and after 16 weeks at a similar time of day, and blood fractions were immediately separated and stored at −80 °C.

#### EPIC-Potsdam cohort

The EPIC-Potsdam cohort study is a prospective cohort study that recruited 27,548 participants (16,644 women and 10,904 men of primarily Middle European ancestry, age range: 35–65 years) from the general population of Potsdam, Germany, and the surrounding geographical area from 1994 to 1998. Follow-up occurred every 2–3 years by mailed questionnaires and, if necessary, by telephone. Response rates ranged between 90% and 96% per follow-up round. The study protocol was approved by the ethics committee of the Medical Society of the State of Brandenburg, Germany, and all participants provided a statement of written informed consent before enrollment.

Incident CVD was defined as incidence of primary nonfatal and fatal MI and stroke (International Statistical Classification of Diseases and Related Health Problems (ICD)-10 codes: I21 for acute MI, I63.0 to I63.9 for ischemic stroke, I61.0 to I61.9 for intracerebral hemorrhage, I60.0 to I60.9 for subarachnoid hemorrhage and I64.0 to I64.9 for unspecified stroke). Incidence of CVD was captured by participants’ self-reports or based on information from the death certificates, which were validated by contacting the treating physicians. Inquired information included ICD-10 code, date of occurrence and further information on symptoms and diagnosis criteria. For MI, diagnostic criteria included clinical symptoms, electrocardiograms, cardiac enzymes and known coronary heart disease. For stroke, diagnosis was based on anamnesis, clinical symptoms, computed tomography/magnetic resonance tomography, angiogram, lumbar puncture, echocardiogram, Doppler and electrocardiogram plus imaging techniques if available. Participants with silent cardiovascular events that had not been documented within 28 days after occurrence were excluded as nonverifiable cases from all analyses.

Information on incidence of T2D was systematically acquired through self-report of a diagnosis, T2D-relevant medication or dietary treatment due to T2D diagnosis during follow-up. Additionally, death certificates and information from tumor centers, physicians or clinics that provided assessments for other diagnoses were screened for indication of incident T2D. For participants who were classified as potential cases based on that information, a standard inquiry form was sent to the treating physician. Only physician-verified cases with a diagnosis of T2D (ICD-10 code E11) and a diagnosis date after the baseline examination were considered confirmed incident cases of T2D.

Nested case–cohorts were constructed for efficient study of molecular phenotypes. From all participants who provided blood at baseline (*n* = 26,437), a random sample (subcohort, *n* = 1,262) was drawn, which served as a common reference population for both endpoints. For each endpoint, all incident cases that occurred in the full cohort until a specified censoring date were included in the analysis. After excluding prevalent cases of the respective outcomes, the analytical sample for T2D comprised 1,886 participants, including 775 incident cases (26 cases in the subcohort), and the analytical sample for CVD comprised 1,671 participants, including 551 incident cases (28 cases in the subcohort). Follow-up was defined as the time between enrollment and study exit determined by diagnosis of the respective disease, death, dropout or final censoring date, whichever came first. Endpoint-specific censoring dates were 30 November 2006 for stroke and MI and 31 August 2005 for T2D.

Anthropometric and blood pressure measurements were conducted according to a standardized protocol^65,66^. Information on lifestyle and education was obtained using computer-assisted personal interviews. These included information on recreational physical activity, smoking status, average alcohol intake and educational attainment. Participants were categorized as hypertensive at study baseline if they had a systolic blood pressure of ≥140 mmHg, diastolic blood pressure of ≥90 mmHg, reported prior diagnosis of hypertension or current antihypertensive medication use. At baseline, trained study personnel obtained 30 ml of peripheral venous blood from each participant. Blood was partitioned into serum, plasma (with 10% of total volume citrate) and blood cells and was subsequently separately stored in tanks of liquid nitrogen at −196 °C or in deep freezers at −80 °C until the time of analysis. Plasma samples, from which aliquots were drawn for the lipidomics measurements in 2016, were never or only once thawed and refrozen during storage (93 samples were defrosted and refrozen once for aliquoting for unrelated analysis).

Plasma concentrations of standard blood lipids (total cholesterol, HDL-C, triglycerides, HbA_1c_, glucose and hsCRP) were measured at the Department of Internal Medicine, University of Tübingen, with an automatic ADVIA 1650 analyzer (Siemens Medical Solutions) in 2007. All biomarker measurements conducted in plasma, including the lipidomics measurements (detailed below), were corrected for the dilution introduced by citrate volume to improve comparability with concentrations measured in EDTA-plasma reported in the literature. Laboratory measurements were conducted by experienced technical personnel following the manufacturer’s instructions. Single imputation based on linear regression was used to impute missing covariate information (participants with missing data for: waist circumference, *n* = 2; BMI, *n* = 12; standard blood lipids (triglycerides, HDL-C and triglycerides), *n* = 82; and blood pressure, *n* = 148).

#### NHSs

The NHS recruited 121,701 female nurses aged 30–55 years in 1976 (ref. ^67^). A subset of 32,826 nurses provided blood samples in 1989 or 1990, of whom 18,743 provided a second blood sample in 2000 or 2001. The NHSII cohort was established in 1989 and recruited 116,429 female nurses aged 25–42 years. In NHSII, blood samples from 29,611 participants were collected between 1996 and 1999. The standardized blood collection procedure is described elsewhere^37^. Participants reported their usual intake of a standard portion of each item in the FFQ (frequency ranging from never to more than six times per day) during the past year every 4 years. The reproducibility and validity of the FFQ has been extensively documented^68–70^. The NHSs were approved by the Human Research Committee at the Brigham and Women’s Hospital, Boston, MA, and participants provided written informed consent.

We computed the intake of individual nutrients by multiplying the frequency of consumption of each food by the nutrient content of the specified portion based on food composition data from the US Department of Agriculture and data from manufacturers. Intake of carbohydrate, fat and protein was expressed as nutrient densities (that is, percent energy)^71^. In a validation study comparing energy-adjusted macronutrient intake assessed by the FFQ with four 1-week diet records, the Pearson correlation coefficients were 0.61 for total carbohydrates, 0.52 for total protein and 0.54 for total fat^70^.

Participants who reported a stroke were asked for permission to review their medical records. For both nonfatal and fatal strokes, available medical records related to the clinical event, such as imaging and autopsy reports, were reviewed by physicians who were blind to participant risk factor status. Strokes were defined according to the National Survey of Stroke criteria and were classified as ischemic or hemorrhagic^72,73^. The ischemic stroke lipidomics case–control study in the NHS/NHSII cohorts used in our analyses included 968 participants with lipidomics data to construct the rMLS (484 case–control pairs). Matching factors included age, fasting, smoking status, race, ethnicity and season of blood collection.

In NHS/NHSII cohorts, T2D incidence was detected based on self-reported diagnosis and was confirmed by a validated supplementary questionnaire^74^. Before 1998, confirmation of T2D incidence relied on the National Diabetes Data Group criteria and from 1998 onward relied on the American Diabetes Association diagnostic criteria. Validation studies in the NHS have demonstrated the validity of the supplementary questionnaires to adjudicate T2D diagnosis, showing that more than 97% of participants with self-reported T2D detected by questionnaires were reconfirmed through medical record review by endocrinologists blinded to questionnaire information^74,75^.

We also designed a 1:1-matched nested case–control study for lipidomics and T2D. Matching factors were age, race, ethnicity and season of blood collection. The T2D case–control study in NHS included 1,456 participants (728 matched case–control pairs) with baseline lipidomics data to construct the rMLS. A subset of case–control pairs had repeated lipidomics data approximately 10 years apart to construct the rMLS based on fasting (≥8 h) blood samples from both times (1989/1990 and 2000/2001). In the repeated blood sampling study, all participants remained diabetes free until after the second blood collection, and all incident T2D cases occurred between 2002 and 2008.

The study protocols were approved by the Institutional Review Boards of Brigham and Women’s Hospital and Harvard T.H. Chan School of Public Health. Participants’ completion of questionnaires was considered as implied consent.

#### PREDIMED trial

The PREDIMED study was a multicenter dietary intervention trial with 7,447 participants in three intervention arms and demonstrated cardiometabolic risk reduction by a Mediterranean diet intervention (www.predimed.es; ISRCTN registry: ISRCTN35739639)^33,76^. The PREDIMED trial received ethical approval from the Institutional Review Board of the Hospital Clinic in Barcelona, Spain, 16 July 2002. The PREDIMED trial inclusion criteria were either prevalence of T2D or prevalence of three or more major cardiovascular risk factors (smoking, dyslipidemia, hypertension and adiposity). Besides the low-fat diet control group, the Mediterranean diet intervention included two arms (one particularly high in extra virgin olive oil and the other particularly high in tree nut intake) that we pooled into one Mediterranean diet group for our primary analyses. Preintervention blood samples were taken after an overnight fast by trained study personnel according to a standard protocol and fractioned, and the EDTA-plasma was stored at −80 °C in deep freezers.

The PREDIMED T2D case–cohort study with available lipidomics data comprised 694 randomly selected participants (approximately 20% of participants) who fulfilled inclusion criteria, that is, no prevalent T2D at recruitment and available plasma samples and all incident T2D cases during a median of 3.8 years of intervention (*n* = 251; per case–cohort design 53 incident T2D cases were randomly included in the subcohort). The analytical sample was restricted to participants with complete data on lipid metabolites in the rMLS (*n* = 678, including 211 participants with incident T2D). Of those, 468 participants (including 148 participants with subsequent T2D incidence) had additional plasma samples and lipidomics profiles from 1 year after recruitment.

The PREDIMED CVD case–cohort study with lipidomics data comprised 791 randomly selected participants with available plasma samples at recruitment (approximately 10% of the eligible participants) and all incident CVD cases during a median of 3.8 years of intervention (*n* = 231). After excluding participants with missing rMLS lipid metabolite values, the analytical sample comprised 871 participants, including 215 participants with incident CVD. Of those, 736 participants (including 136 participants with subsequent CVD incidence) had additional plasma samples and lipidomics profiles from 1 year after recruitment. The study protocols were approved by the Institutional Review Boards at all study locations (PREDIMED) and the Harvard T.H. Chan School of Public Health (PREDIMED case–control subproject). All participants gave written informed consent.

#### LIPOGAIN-2 trial

The LIPOGAIN-2 study was a 12-week, double-blind, parallel-group randomized trial focusing on overweight individuals. In this manuscript, only the first phase of the trial, consisting of an 8-week overfeeding period, was used.

Participants aged between 20 and 55 years with a BMI ranging from 25 to 32 kg m^–2^ were eligible. Exclusion criteria were diabetes (fasting glucose of >7 mM on two occasions) or liver disease, pregnancy, lactation, alcohol abuse, claustrophobia, abnormal clinical chemistry test results, use of drugs influencing energy metabolism, use of omega-3 supplements or extreme diets, regular heavy exercise (>3 h per week), intolerance to gluten, egg or milk protein and implanted metals. Participants were required to fast overnight for 10 to 12 h and avoid physical exercise and alcohol for 48 h before measurements were taken.

The trial took place at Uppsala University Hospital in Uppsala, Sweden, from August 2014 to June 2015. Participants were assigned to groups through a computer-generated list, which was prepared by a statistician not involved in the study, and stratified for sex, age and BMI. This study is registered on www.clinicaltrials.gov under the identifier NCT02211612 and was conducted in accordance with the Declaration of Helsinki. All participants provided written informed consent before inclusion, and the study was approved by the Regional Ethical Review Board in Uppsala (Dnr 2014/186).

In total, 61 participants were randomized to receive muffins made with either refined sunflower oil, which is high in PUFAs (specifically linoleate 18:2n-6), or refined palm oil rich in SFAs (mainly palmitate 16:0) for 8 weeks. Participant body weight was monitored weekly when they visited the clinic to receive their muffins, which were prepared in large batches under controlled conditions in a metabolic kitchen at Uppsala University. These muffins, identical in composition except for the type of fat, were added to the participants’ regular diets to be eaten at any time of the day. Their number was adjusted weekly by plus or minus one muffin per day based on the rate of weight gain, with the goal being an average weight gain of 3% (equivalent to about 2.9 ± 0.5 muffins or approximately 40 g of oil per day). The muffins comprised 51% fat, 44% carbohydrates and 5% protein by energy percentage. One participant was removed due to missing sphingolipid measurements.

### Lipidomics profiling

#### DIVAS trial and EPIC-Potsdam cohort

Lipidomics analysis was performed with Metabolon’s Complex Lipid Panel for the EPIC-Potsdam cohort and the DIVAS trial separately. In brief, the platform generates concentrations of molecular species and nearly complete fatty acid composition per lipid class in plasma. The lipid fraction is extracted with methanol:dichloromethane, concentrated under nitrogen and reconstituted in ammonium acetate dichloromethane:methanol (BUME extraction). Extracts are then infused into the ionization source of a Sciex SelexION-5500 QTRAP mass spectrometer operated in multiple reaction monitoring mode with positive/negative switching. Lipid classes are subsequently separated by differential mobility spectrometry. Using 1,100 multiple reaction monitorings, lipid mass and characteristic fragments are determined with the help of more than 50 isotopically labeled internal standards that are simultaneously introduced with the biological sample. Molecular species are quantified by taking the ratio of the signal intensity of each target compound to that of its assigned internal standard and multiplying by the concentration of internal standard added to the sample^77^.

The Complex Lipid Panel produced measurements for 14 lipid classes (cholesteryl esters, monoglycerides, ceramides, dihydroceramides, lactosylceramides, hexosylceramides, sphingomyelins, lysophosphatidylethanolamines, lysophosphatidylcholines, diglycerides, triglycerides, phosphatidylcholines, phosphatidylethanolamines and phosphatidylinositol). For phosphatidylethanolamines, species from the two subclasses phosphatidylethanolamine ether and phosphatidylethanolamine plasmalogen were detected. Measured concentrations of molecular species were used to calculate within-class fatty acid sums (summing all concentrations of molecular species containing a specific fatty acid within a lipid class). Within-class fatty acid sums are synonymous with molecular species level in lipid classes containing only one reported variable fatty acid per molecule (one-fatty-acid-containing classes: cholesteryl esters, monoglycerides, ceramides, dihydroceramides, lactosylceramides, hexosylceramides, sphingomeylins, lysophosphatidylethanolamines and lysophosphatidylcholines).

For comparability with the species-level lipidomics in the PREDIMED trial and NHS/NHS2 cohorts (see below), we further calculated the species level for those classes with more than one fatty acid per molecule (that is, diglycerides, triglycerides, phosphatidylcholines, phosphatidylethanolamines and phosphatidylinositol) by summing all species with the same total atomic mass and degree of saturation of the contained fatty acids (that is, isobaric species). We used the updated shorthand notations from the LIPIDMAPS initiative where applicable^78^. We only refer to the shorthand notations of fatty acids for brevity. According to the manufacturer, the median coefficient of variation of species at a 1 µM concentration in serum or plasma was approximately 5%. Several lipid species had higher percentages of missing values because they were likely below the lower limit of quantification. Lipid species with more than 70% missing values were excluded, while missing values in the remaining lipid species were imputed using the ‘Quantile Regression Imputation of Left-Censored data’ approach from the R package imputeLCMD (https://CRAN.R-project.org/package=imputeLCMD).

#### NHS/NHSII cohorts and the PREDIMED trial

At the Broad Institute, plasma polar and nonpolar lipids were identified using a Shimadzu Scientific Instrument Nexera x2 U-HPLC system, which was linked to a Thermo Fisher Scientific Exactive Plus Orbitrap mass spectrometer. Lipids were extracted from the plasma (10 µl) using 190 µl of isopropanol that had 1,2-didodecanoyl-*sn*-glycero-3-phosphocholine as an internal standard, supplied by Avanti Polar Lipids. After centrifugation (10 min, 9,000*g*, room temperature), the supernatants (2 µl) were directly injected onto a 100 × 2.1 mm ACQUITY BEH C8 column (1.7 µm) from Waters. The column was flushed isocratically at a flow rate of 450 µl min^–1^ for 1 min at 80% of mobile phase A (95:5:0.1 (vol/vol/vol) of 10 mmol l^–1^ ammonium acetate:methanol:acetic acid), succeeded by a linear gradient to 80% of mobile phase B (99.9:0.1 (vol/vol) methanol:acetic acid) for 2 min and a linear gradient to 100% mobile phase B over 7 min and finally maintained at 100% mobile phase B for 3 min.

Mass spectrometry analyses were performed in positive ion mode using electrospray ionization and full scan analysis over *m*/*z* 200–1,100 at a resolution of 70,000 and a data acquisition rate of 3 Hz. The following other mass spectrometry parameters were used: ion spray voltage at 3.0 kV, capillary and probe heater temperature at 300 °C, sheath gas at 50, auxiliary gas at 15 and S-lens RF level at 60. Progenesis QI software (NonLinear Dynamics) was used to process raw data for feature alignment, nontargeted signal detection and signal integration. Targeted processing of a subset of lipids was conducted using TraceFinder software (version 3.2; Thermo Fisher Scientific). Lipids were characterized by their headgroup, overall acyl carbon content and total acyl double bond content^79^. The Broad Institute metabolomics data in NHS/NHSII were measured in several case–control studies. Within each case–control study, lipid species with more than 70% missing values were excluded, whereas missing values in remaining lipid metabolites were imputed with half the minimal measured value. Due to the platform evolution in the NHS/NHSII cohorts, some metabolite levels were not measured in specific case–control studies. For calculation of the rMLS, nonmeasured values of specific metabolites in specific case–control studies were substituted with the median of all measured values across the whole dataset (only applicable to the rMLS diet substitution models in the NHS/NHSII cohorts).

#### LIPOGAIN-2 trial

Sphingolipids from serum were extracted using butanol–methanol methods^80,81^. Sphingolipids were detected and quantified using ultraperformance liquid chromatography/tandem mass spectrometry, as previously described^82^.

### Statistical analysis

All lipidomics variables in all study samples were log transformed.

#### DIVAS diet effect

We assessed the difference in postintervention within-class fatty acid sum concentrations between the SFA-rich and UFA-rich diets via linear regression models with trial arm coded as an indicator variable (SFA-rich diet as a reference) and adjusted for respective baseline concentrations in addition to age, BMI and sex. All lipids that were statistically significantly different between the diets after controlling for an FDR^83^ at 5% were used for calculating the MLS (Supplementary Tables 10 and 11). Using the estimated intervention effects as weights, we calculated the MLS in the DIVAS trial and, again, used linear regression to estimate baseline-adjusted differences in MLS between the diets. For the analyses of sphingolipids, sphingolipid score and apolipoprotein B in the LIPOGAIN-2 trial, we used the same approach as in the DIVAS trial. The models were similarly adjusted for age, sex and BMI.

#### Calculation of the MLS in the EPIC-Potsdam cohort

Using the estimated intervention effects as weights, we calculated the MLS in the EPIC-Potsdam cohort and divided the score by the observed diet effect on the MLS in the DIVAS trial so that one unit increase in the MLS corresponds to the magnitude of the DIVAS diet intervention effect. Like the above approach, we estimated the diet effect on other risk biomarkers (HbA_1c_, fasting glucose, total triglycerides, HDL-C, non-HDL-C and hsCRP) and applied the respective observed effect as a scale for the hypothetical DIVAS intervention effect in the EPIC-Potsdam cohort.

#### Risk associations with CVD and T2D in the EPIC-Potsdam cohort

We assessed the association between MLS and incident CVD and T2D with Cox proportional hazards models. The case–cohort design was accounted for by assigning weights as proposed by Prentice. Age was the underlying time variable, with entry time as age at baseline and exit time as age at event or censoring. The fully adjusted model included age (years), sex, waist circumference (cm), height (cm), leisure-time physical activity (average h per week), highest achieved education level (three categories: primary school, secondary school/high school and college/higher education degree), fasting status at blood draw (three categories: overnight fast, only drink and unfasted), total energy intake (g day^–1^), blood pressure (systolic and diastolic; mmHg), smoking status (four categories: never, former, current smoker (<20 U day^–1^) and current smoker (≥20 U day^–1^)), alcohol intake (six sex-specific categories: none, low, moderately low, moderately high, high and very high), antihypertensive medication (yes/no), lipid-lowering medication (yes/no) and acetylsalicylic acid medication (yes/no) as covariates. Models for CVD were additionally adjusted for prevalent T2D. To check if the presentation of stratified results was warranted, we tested the potential for effect measure modification by sex by including MLS × sex interaction terms into the multivariable-adjusted model.

#### Derivation of the rMLS

The rMLS was constructed with the same weights as were used in the EPIC-Potsdam cohort; however, those lipids that were not available in the Broad Institute lipidomics data in the NHS/NHSII cohorts and PREDIMED trial were either skipped or, where possible, imputed using regression weights from the EPIC-Potsdam cohort. In detail, the Broad Institute lipidomics datasets available in the NHS/NHSII cohorts and the PREDIMED trial offer species-level lipidomics in those lipid classes that contain more than one fatty acid residue per molecule, whereas the platform used in the EPIC-Potsdam cohort and the DIVAS trial generated resolution down to the molecular species level, indicating all fatty acid residues per molecule (with the exception of triglycerides). We calculated species levels in the EPIC-Potsdam cohort and used these to predict within-class fatty acid sums. These lipid species-specific weights were then applied to generate a predicted value of the missing lipid variable in the PREDIMED trial and the NHS/NHSII cohorts, where possible.

#### Diet substitution models, risk associations and analysis of change in NHS/NHSII

Diet and lipidomics profiles were available from 10,894 women in the NHS (*n* = 7,479) and NHSII (*n* = 3,415) cohorts. For macronutrient substitution modeling, we used the average of the macronutrient intakes derived from the two FFQs closest to the blood collection that was used in the dietary substitution analyses (NHS cohort: 1986 and 1990; NHSII cohort: 1995 and 1999). We then included all dietary macronutrient variables (as percent total energy) except for saturated fat in a linear model with the variance standardized MLS as outcome, adjusting for total energy intake excluding alcohol (kcal day^–1^), alcohol intake (g day^–1^), BMI (kg m^–2^), age (years) and diet quality (AHEI without alcohol points). Macronutrient intake was scaled to 8% of total energy. Therefore, effect estimates from this linear model can be interpreted as the association of substituting 8% of total energy from SFAs with 8% of total energy from other macronutrients. Conditional logistic regression models were used to assess the associations of the rMLS with the risk of developing stroke and T2D.

We further assessed the correlation of the rMLS with established diet quality indices, including LCDs^84^, the aMed^85^ and the AHEI^86^. For the general LCD, participants were divided into 11 strata based on percentage of energy from each total fat, protein and carbohydrates. Points were assigned descending from 10 for the highest stratum in fat and protein to 0 for the lowest. For carbohydrates, scoring was reversed, with the lowest intake receiving 10 points and the highest receiving 0. We applied the same methodology to compute two additional LCD scores: one animal based and one vegetable based. The animal-based LCD score was based on the percentage of energy derived from carbohydrates, animal protein and animal fat. Conversely, the vegetable-based LCD score was calculated from the energy percentages from carbohydrates, vegetable protein and vegetable fat^84^.

The aMed score, adapted from Trichopoulou et al.^87^, includes vegetables (excluding potatoes), fruits, nuts, whole grains, legumes, fish and the ratio of monounsaturated to saturated fats along with red and processed meats and alcohol. Participants scoring above the median intake in these categories received 1 point, except for red and processed meats where scoring below the median earned a point; all others received 0. Alcohol intake scoring awarded 1 point for daily consumption between 5 and 15 g. The aMed score ranges from 0 to 9, with higher scores indicating greater adherence to the Mediterranean diet^85^.

The AHEI was developed after a comprehensive literature review and consultations with nutrition researchers to identify dietary factors consistently linked with a reduced risk of chronic diseases in clinical and epidemiological research. Beneficial AHEI components include vegetables, fruits, whole grains, nuts, legumes, long-chain omega-3 PUFAs and total PUFAs, whereas adverse components comprise sugar-sweetened beverages, red and processed meats, *trans*-fats and sodium. Moderate alcohol consumption scores highest, with high consumption scoring lowest. Each AHEI component is rated from 0 (worst) to 10 (best), resulting in a total score ranging from 0 (no adherence) to 110 (perfect adherence)^86^.

Risk associations with stroke and T2D in the nested 1:1-matched case–control studies were assessed with conditional logistic regressions adjusted for age, BMI, alcohol intake, diet quality and smoking. Analyses on 10-year change in MLS were further adjusted for status after 10 years of these variables (except age).

#### Risk associations and interaction analyses in the PREDIMED trial

We used Prentice-weighted Cox proportional hazards regression to assess the association between the rMLS and the risk of incident disease endpoints in PREDIMED. The interaction analyses were performed in the subsamples with two lipidomics profiles (preintervention and 1-year into the intervention). The interaction model contained a three-way interaction term between Mediterranean diet intervention and the repeated rMLS measurements (preintervention rMLS × Mediterranean diet intervention × 1-year intervention rMLS) along with the main effect terms and were adjusted for age and sex. The results of the interaction analyses informed the subsequent stratified analyses according to the Mediterranean diet intervention. The Cox models in the intervention strata were adjusted for age, sex and preintervention BMI.

#### Network and cluster analysis in the EPIC-Potsdam cohort

We estimated a network model of conditional dependencies, where edges represent covariance between two lipids that could not be explained by adjustment for any subset of other lipids. To this end, we applied an order-independent implementation of the causal structure learning PC algorithm^88^. The resulting network graphically encoded the family of causal models that could have generated the observed conditional independence structure, that is, the skeleton of the data-generating directed acyclic graph. Within this network, we identified clusters of lipids using the Louvain modularity detection algorithm. The Louvain method is a fast heuristic algorithm for detecting communities in large networks by optimizing modularity. It iteratively merges nodes into communities to maximize within-community links compared to between-community links^38^.

We then calculated cluster-specific lipid scores using the same weights as for the full MLS and associated the resulting scores with risk of cardiometabolic diseases in the same way as the full MLS. We furthermore applied the NetCoupler algorithm (netcoupler.github.io/NetCoupler/) to identify those lipid–disease connections that could not be attributed to the influence of related MLS lipids. The algorithm uses the conditional independence network to detect links between individual lipids and disease incidence that could not be explained by confounding influences through other lipids. By definition, at least one subset of direct neighbors is sufficient to block confounding from the whole network. However, sufficient adjustment sets cannot be unambiguously read from the graph because the edges are not directed. Therefore, the NetCoupler algorithm iterates for each lipid through adjustment for all possible combinations of direct network neighbors. A lipid is then only classified as a direct effector if the association with disease incidence is robust across all these submodels^39,40^.

### Software

All analyses were performed using R (version 4.3.0). Further information on used R packages is reported in Supplementary Table 13.

### Reporting summary

Further information on research design is available in the Nature Portfolio Reporting Summary linked to this article.

## Online content

Any methods, additional references, Nature Portfolio reporting summaries, source data, extended data, supplementary information, acknowledgements, peer review information; details of author contributions and competing interests; and statements of data and code availability are available at 10.1038/s41591-024-03124-1.

## Supplementary information

Reporting Summary

Supplementary Tables Supplementary Tables 1–13.

## Source data

Source Data Fig. 2 Statistical source data.

Source Data Fig. 3 Statistical source data.

Source Data Fig. 4 Statistical source data.

Source Data Fig. 5 Statistical source data.

Source Data Fig. 6 Statistical source data.

Source Data Extended Data Fig. 1 Statistical source data.

Source Data Extended Data Fig. 2 Statistical source data.

Source Data Extended Data Fig. 3 Statistical source data.

Source Data Extended Data Fig. 5 Statistical source data.

Source Data Extended Data Fig. 6 Statistical source data.

Source Data Extended Data Fig. 7 Statistical source data.

Source Data Extended Data Fig. 8 Statistical source data.

Source Data Extended Data Fig. 9 Statistical source data.

Source Data Extended Data Fig. 10 Statistical source data.

## Data Availability

The research data supporting the findings of this study consist of sensitive human information derived from the following contributing studies: the DIVAS trial, LIPOGAIN-2 trial, EPIC-Potsdam cohort, NHS/NHSII cohorts and PREDIMED trial. To ensure the confidentiality and protection of participant data, all datasets are governed by an approved data access policy, which adheres to data security and ethical considerations. Access to these datasets is available for research and validation purposes, subject to adherence to institutional data security protocols. According to standard controlled access procedures, applications to use resources from the participating studies will be reviewed by External Collaborations and Scientific Steering Committees to verify that the proposed use maintains the protection of the privacy of the participants and the confidentiality of the data in EPIC-Potsdam (convening monthly), NHSI and NHSII (convening biweekly) and PREDIMED (convening monthly) and the principal study investigators in DIVAS (J.A.L.) and LIPOGAIN-2 (U.R.). Study-specific contact and data access information can be obtained from the corresponding authors or the following sources: DIVAS trial (https://research.reading.ac.uk/ifnh/cases/milk-dairy-consumption-risk-cardiovascular-diseases-cause-mortality/), EPIC-Potsdam cohort (https://www.dife.de/en/research/cooperations/epic-study/), NHS and NHSII cohorts (https://nurseshealthstudy.org/) and PREDIMED trial (http://www.predimed.es/). Source data are provided with this paper.

## Code availability

Code is available via Zenodo at 10.5281/zenodo.11412029 (ref. ^89^).
