# Supplementary material for: Lipidome changes due to improved dietary fat quality inform cardiometabolic risk reduction and precision nutrition
Source: Nat Med. 2024 Jul 11;30(10):2867–77. doi: 10.1038/s41591-024-03124-1 (PMC11485259; doi:10.1038/s41591-024-03124-1)
Supplement: Supplementary file 1 — Reporting Summary [file 41591_2024_3124_MOESM1_ESM.pdf]

nature portfolio

Corresponding author(s): Clemens Wittenbecher

Last updated by author(s): May 28, 2024

Reporting Summary

Nature Portfolio wishes to improve the reproducibility of the work that we publish. This form provides structure for consistency and transparency in reporting. For further information on Nature Portfolio policies, see our Editorial Policies and the Editorial Policy Checklist.

Statistics

For all statistical analyses, confirm that the following items are present in the figure legend, table legend, main text, or Methods section.

n/a	Confirmed	
☐	☒	The exact sample size (*n*) for each experimental group/condition, given as a discrete number and unit of measurement
☐	☒	A statement on whether measurements were taken from distinct samples or whether the same sample was measured repeatedly
☐	☒	The statistical test(s) used AND whether they are one- or two-sided<br>*Only common tests should be described solely by name; describe more complex techniques in the Methods section.*
☐	☒	A description of all covariates tested
☐	☒	A description of any assumptions or corrections, such as tests of normality and adjustment for multiple comparisons
☐	☒	A full description of the statistical parameters including central tendency (e.g. means) or other basic estimates (e.g. regression coefficient) AND variation (e.g. standard deviation) or associated estimates of uncertainty (e.g. confidence intervals)
☐	☒	For null hypothesis testing, the test statistic (e.g. F, t, r) with confidence intervals, effect sizes, degrees of freedom and P value noted<br>*Give P values as exact values whenever suitable.*
☒	☐	For Bayesian analysis, information on the choice of priors and Markov chain Monte Carlo settings
☒	☐	For hierarchical and complex designs, identification of the appropriate level for tests and full reporting of outcomes
☐	☒	Estimates of effect sizes (e.g. Cohen's *d*, Pearson's *r*), indicating how they were calculated

Our web collection on statistics for biologists contains articles on many of the points above.

Software and code

Policy information about availability of computer code

Data collection	No software was used for data collection since all analyses rely on secondary and post-hoc data.
Data analysis	R (version 4.3.0; R Core Team, 2023)<br>NetCoupler (version 0.1.0.9000; Johnston L, Wittenbecher C, 2024)<br>imputeLCMD (version 2.1; Lazar C, Burger T, 2022)<br>survival (version 3.5.0; Therneau T, 2023)<br>tidyverse (version 2.0.0; Wickham H et al., 2019)<br>patchwork (version 1.1.2; Pedersen T, 2022)<br>Custom code: https://zenodo.org/doi/10.5281/zenodo.11412029

For manuscripts utilizing custom algorithms or software that are central to the research but not yet described in published literature, software must be made available to editors and reviewers. We strongly encourage code deposition in a community repository (e.g. GitHub). See the Nature Portfolio guidelines for submitting code & software for further information.

Data

Policy information about availability of data

All manuscripts must include a data availability statement. This statement should provide the following information, where applicable:

- Accession codes, unique identifiers, or web links for publicly available datasets
- A description of any restrictions on data availability
- For clinical datasets or third party data, please ensure that the statement adheres to our policy

The research data supporting the findings of this study consist of sensitive human information, derived from the contributing studies: DIVAS trial, LIPOGAIN-2 trial, EPIC-Potsdam cohort, NHS/NHSII cohorts, and the PREDIMED trial. To ensure the confidentiality and protection of participant data, all datasets are governed by an approved data access policy, which adheres to data security and ethical considerations. Access to these datasets is available for research and validation purposes, subject to adherence to institutional data security protocols. . According to standard controlled access procedures, applications to use resources from the participating studies will be reviewed by External Collaborations and Scientific Steering Committees to verify that the proposed use maintains the protection of the privacy of participants and the confidentiality of the data in EPIC-Potsdam (convening monthly), NHS I and NHS II (convening biweekly), and PREDIMED (convening monthly), and the principal study investigators in DIVAS (JAL) and LIPOGAIN-2 (UR).Study-specific contact and data access information can be obtained from the corresponding author or the following sources: DIVAS trial: https://research.reading.ac.uk/ifnh/cases/milk-dairy-consumption-risk-cardiovascular-diseases-cause-mortality/; EPIC-Potsdam cohort: https://www.dife.de/en/research/cooperations/epic-study/; NHS & NHSII cohorts: https://nurseshealthstudy.org/; PREDIMED trial: http://www.predimed.es/.

Research involving human participants, their data, or biological material

Policy information about studies with human participants or human data. See also policy information about sex, gender (identity/presentation), and sexual orientation and race, ethnicity and racism.

Reporting on sex and gender	We use sex as biological variable in multi-variable adjusted models and interaction analyses and report this accordingly.
Reporting on race, ethnicity, or other socially relevant groupings	As described in the methods, the large majority of participants were White. Geographical and occupational recruitment areas are described in the Methods section. E.g., White from Middle European descent (EPIC-Potsdam), Matching factors were age, race, ethnicity, and time of blood collection (NHS), etc.
Population characteristics	The EPIC-Potsdam study is a prospective cohort study that recruited 27,548 participants (16,644 women and 10,904 men, White from Middle European descent, age-range: 35-65 years) from the general population of Potsdam, Germany, and the surrounding geographical area from 1994 to 1998. Participants were then actively followed-up every 2-3 years, by mailed questionnaires and, if necessary, by telephone. Response rates ranged between 90% and 96% per follow-up round.<br><br>The NHS recruited 121,701 female nurses aged 30–55 years in 197642. A subset of 32,826 nurses provided blood samples in 1989 or 1990, of whom 18,743 provided a second blood sample in 2000 or 2001. The NHSII was established in 1989 and recruited 116,429 female nurses aged 25–42 years. In NHSII, blood samples from 29,611 participants were collected between 1996 and 1999.<br><br>DIVAS [is] a 16-week randomized controlled trial (registered at www.clinicaltrials.gov as NCT01478958). This study recruited men and women, aged between 21-60 years and with estimated moderate CVD risk which were randomized to either one of three isoenergetic diets: rich in saturated FAs (SFA), rich in monounsaturated FAs (MUFA), or rich in mixed unsaturated fatty acids (UFA) including both MUFA and n–6 polyunsaturated FAs (PUFA).<br><br>The PREDIMED study was a multicenter dietary intervention trial with 7447 participants in 3 intervention arms and demonstrated cardiometabolic risk reduction by a Mediterranean diet intervention (www.predimed.es, ISRCTN registry: ISRCTN35739639). The PREDIMED trial inclusion criteria were either prevalent type 2 diabetes or prevalence of three or more major cardiovascular risk factors (smoking, dyslipidemia, hypertension, adiposity).<br><br>LIPOGAIN-2<br>Participants aged between 20 and 55 years with a BMI ranging from 25 to 32 kg/m² were eligible. Exclusion criteria were: diabetes (fasting glucose >7 mM at two occasions) or liver disease; pregnancy; lactation; alcohol abuse; claustrophobia; abnormal clinical chemistry test results; use of drugs influencing energy metabolism; use of omega-3 supplements or extreme diets; regular heavy exercise (>3 h/week); intolerance to gluten, egg, or milk protein; and implanted metals. Participants were required to fast overnight for 10 to 12 hours and avoid physical exercise and alcohol for 48 hours before measurements were taken.
Recruitment	EPIC-Potsdam<br>Participants were actively recruited from the general population of Potsdam, Germany, and the surrounding geographical area from 1994. Potential participants were randomly selected from registries and contacted.<br><br>NHS<br>Married registered nurses, aged 30 to 55 in 1976, who lived in the 11 most populous states, and whose nursing boards agreed to supply NHS with their members' names and addresses, were eligible to be enrolled in the cohort if they responded to the NHS baseline questionnaire<br><br>DIVAS Men and women were recruited from the Reading area in the United Kingdom via advertisement.

PREDIMED high-risk patients

LIPOGAIN-2 Participants were recruited via advertisements.

Ethics oversight

The DIVAS study was conducted according to the guidelines of the Declaration of Helsinki and favorable ethical opinion for conduct was given by the West Berkshire Local Research Ethics Committee (09/H0505/56) and the University of Reading Research Ethics committee (09/40). All participants provided written informed consent before participating. EPIC-Potsdam: The study protocol was approved by the ethics committee of the Medical Society of the State of Brandenburg, Germany, and all participants provided a statement of written informed consent prior to enrollment. NHS/NHSII: The study protocols were approved by the institutional review boards of Brigham and Women's Hospital and Harvard T.H. Chan School of Public Health. Participants' completion of questionnaires was considered as implied consent. The PREDIMED trial received ethical approval of the Institutional Review Board of the Hospital Clinic at Barcelona, Spain, 16/07/2002. LIPOGAIN-2 was conducted in accordance with the Declaration of Helsinki. All subjects provided written, informed consent before inclusion, and the study was approved by the Regional Ethical Review Board in Uppsala (Dnr 2014/186).

Note that full information on the approval of the study protocol must also be provided in the manuscript.

Field-specific reporting

Please select the one below that is the best fit for your research. If you are not sure, read the appropriate sections before making your selection.

☒ Life sciences ☐ Behavioural & social sciences ☐ Ecological, evolutionary & environmental sciences

For a reference copy of the document with all sections, see nature.com/documents/nr-reporting-summary-flat.pdf

Life sciences study design

All studies must disclose on these points even when the disclosure is negative.

Sample size

We used trials for post-hoc analyses. Therefore, the sample size calculation for the primary trial outcomes are not relevant for this study. In endpoint analyses, we maximized the statistical power and cost efficiency by using case cohort-designs (EPIC-Potsdam, PREDIMED) and case-control designs (NHS, NHSII). The exact numbers for the various study samples and for each specific analysis are reported in the results and methods sections.

Data exclusions

We used imputation below the limit of detection for missing metabolomics data and excluded participants with missing diet and covariable data. Model-specific exclusions are reported in the results section.

Replication

Disease associations in EPIC-Potsdam are replicated in NHS/NHSII. DIVAS effects on lipids were replicated for sphingolipids in LIPOGAIN-2

Randomization

The DIVAS study, LIPOGAIN-2, and the PREDIMED study are randomized trials. The primary PREDIMED study investigators reported deviations from the randomization protocol at 1 of 11 study centers (https://www.nejm.org/doi/full/10.1056/nejmoa1800389). Re-analysis of the data did not affect the primary outcome results.

Blinding

The DIVAS study was single blinded and the participants were unaware of the fatty acid composition of their intervention diet. All molecular data was generated and processed blinded to the case status and other participant data. LIPOGAIN-2 was doubleblinded. PREDIMED used a complex dietary intervention, which is not possible to fully blind. Blinding is not relevant for observational studies (EPIC-Potsdam, NHS/NHSII)

Reporting for specific materials, systems and methods

We require information from authors about some types of materials, experimental systems and methods used in many studies. Here, indicate whether each material, system or method listed is relevant to your study. If you are not sure if a list item applies to your research, read the appropriate section before selecting a response.

Materials & experimental systems

n/a	Involved in the study	
☒	☐	Antibodies
☒	☐	Eukaryotic cell lines
☒	☐	Palaeontology and archaeology
☒	☐	Animals and other organisms
☐	☒	Clinical data
☒	☐	Dual use research of concern
☒	☐	Plants

Methods

n/a	Involved in the study	
☒	☐	ChIP-seq
☒	☐	Flow cytometry
☒	☐	MRI-based neuroimaging

Clinical data

Policy information about clinical studies

All manuscripts should comply with the ICMJE guidelines for publication of clinical research and a completed CONSORT checklist must be included with all submissions.

Clinical trial registration

DIVAS, www.clinicaltrials.gov as NCT01478958. PREDIMED, ISRCTN registry: ISRCTN35739639. LIPOGAIN-2, www.clinicaltrials.gov as NCT02211612.

Study protocol

DIVAS, https://doi.org/10.3945/jn.114.190645; PREDIMED: https://www.nejm.org/doi/suppl/10.1056/NEJMoa1800389/suppl_file/nejmoa1800389_protocol.pdf; https://www.nejm.org/doi/suppl/10.1056/NEJMoa1800389/suppl_file/nejmoa1800389_appendix.pdf ; LIPOGAIN-2 https://doi.org/10.1210%2Fjc.2019-00160

Data collection

DIVAS.
Study Start, 2010-05. Primary Completion (Actual), 2012-10.
Ages Eligible for Study: 21 Years to 60 Years (Adult)
Enrollment (Actual), 202.

Inclusion Criteria:
Adults should have a relative risk (RR) of > 1.5 of developing cardiovascular disease (CVD) based on presenting with at least one recognised risk factor for CVD:
total cholesterol (TC) > 6.0 mmol/l
HDL cholesterol (HDLC) ≤ 1.0 mmol/l male, ≤ 1.3 mmol/l female
Glucose ≥ 6 mmol/l
Stage 1 hypertension or above i.e. a systolic BP ≥ 140 mmHg, diastolic BP ≥ 90 mmHg
BMI 28-35 kg/m2
waist >102 cm male or > 84 cm female
Adults with a first degree relative with either a history of premature CVD - age of onset younger than 55 y in fathers, sons or brothers or younger than 65 y in mothers, daughters or sisters, or type 2 diabetes.

Exclusion Criteria:
having suffered a myocardial infarction/stroke in the past 12 months
diabetic (diagnosed or fasting glucose > 7 mmol/l) or suffer from other endocrine disorders
suffering from renal or bowel disease or have a history of choleostatic liver or pancreatitis
on drug treatment for hyperlipidaemia, hypertension, inflammation or hypercoagulation
no history of alcohol abuse
planning or on a weight reducing regime
taking any fish oil, fatty acid or vitamin and mineral supplements
pregnant, lactating or planning a pregnancy
smokers

PREDIMED.
Overall study start date, 01/10/2003. Overall study end date, 31/12/2011.
Eligibility
Participant inclusion criteria
Participants are community-dwelling high-risk persons aged 55 to 80 for men and aged 60 to 80 for women, without a history of cardiovascular disease (CHD), who fulfill at least one of the two following criteria:
1. Type 2 diabetes
2. Three or more of the risk factors:
2.1. Current smoker
2.2. Hypertension
2.3. Hypercholesterolemia (low density lipoprotein cholesterol [LDL-cholesterol] more than 160 mg/dl or treatment with hypolipidemic drugs)
2.4. High density lipoprotein cholesterol (HDL-cholesterol) less than 40 mg/dl
2.5. Overweight or obesity (body mass index more than 25 kg/m^2)
2.6. Family history of premature CHD
Participant type: Patient
Age group: Senior. Lower age limit, 55 Years; Upper age limit, 80 Years.
Target number of participants, n = 7500 high-risk participants. Total final enrolment, 7447.

Participant exclusion criteria
1. Previous history of cardiovascular disease
2. Any severe chronic illness
3. Immunodeficiency or human immunodeficiency virus (HIV) positive status
4. Illegal drug use or chronic alcoholism
5. History of allergy to olive oil or nuts
6. Low predicted likelihood of changing dietary habits according to the Prochaska and DiClemente stages of change model

Recruitment start date: 01/10/2003
Recruitment end date: 31/12/2011

LIPOGAIN-2
Overall study start date, 2014-09. Overall study end date, 2015-06.

Inclusion Criteria:
Body mass index 25-32
Age range 30-50

Exclusion Criteria:
- Type 2 diabetes
- Type 1 diabetes
- Kidney disease
- Liver disease
- Abnormal clinical chemistry at screening
- Intense physical exercise > 2 hours per week
- Use of statins or drugs affecting energy metabolism
- Use of extreme diets

Outcomes

DIVAS: Cardiovascular Risk Factors (Lipids, Inflammatory Markers, Indices of Insulin Resistance, Cell Microparticles, Endothelial Progenitor Cells).
PREDIMED. Primary outcome: composite endpoint of cardiovascular death, non-fatal myocardial infarction, and non-fatal stroke. Secondary outcomes: death of any cause and incidence of angina leading to a revascularization procedure, heart failure, diabetes mellitus, cancer, dementia, and other degenerative disorders.
LIPOGAIN-2: Primary outcome: Change in lean tissue mass measured via MRI and air displacement plethysmography; secondary outcomes: Change in hepatic fat content, Change in visceral adipose tissue (VAT), Change in total adipose tissue, Change in pancreatic fat, Change in insulin sensitivity, Change in plasma lipids, Change in circulating inflammation and coagulation markers

Plants

Seed stocks

Report on the source of all seed stocks or other plant material used. If applicable, state the seed stock centre and catalogue number. If plant specimens were collected from the field, describe the collection location, date and sampling procedures.

Novel plant genotypes

Describe the methods by which all novel plant genotypes were produced. This includes those generated by transgenic approaches, gene editing, chemical/radiation-based mutagenesis and hybridization. For transgenic lines, describe the transformation method, the number of independent lines analyzed and the generation upon which experiments were performed. For gene-edited lines, describe the editor used, the endogenous sequence targeted for editing, the targeting guide RNA sequence (if applicable) and how the editor was applied.

Authentication

Describe any authentication procedures for each seed stock used or novel genotype generated. Describe any experiments used to assess the effect of a mutation and, where applicable, how potential secondary effects (e.g. second site T-DNA insertions, mosiacism, off-target gene editing) were examined.
